# The proteomics and phosphoproteomics landscape of melanoma under T cell attack

**DOI:** 10.1016/j.xcrm.2026.102829

**Published:** 2026-05-21

**Authors:** Giulia Franciosa, Agnete W.P. Jensen, Ana Martinez-Val, Ilaria Piga, Marco Donia, Jesper V. Olsen

**Affiliations:** 1 Novo Nordisk Foundation Center for Protein Research, Department of Cellular and Molecular Medicine, Faculty of Health and Medical Sciences, University of Copenhagen, 2200 Copenhagen, Denmark; 2 Cardiovascular Proteomics Laboratory, Centro Nacional de Investigaciones Cardiovasculares Carlos III (CNIC), 28029 Madrid, Spain; 3 Centro de Investigación Biomédica en Red de Enfermedades Cardiovasculares (CIBERCV), 28029 Madrid, Spain; 4 The Functional Genomics Center Zurich (FGCZ), University of Zurich and ETH Zurich, 8057 Zurich, Switzerland; 5 National Center of Cancer Immune Therapy, Department of Oncology, Copenhagen University Hospital - Herlev and Gentofte, 2730 Herlev, Denmark

**Keywords:** proteomics, phosphoproteomics, mass spectrometry, cell signaling, melanoma, TILs, cancer, immunotherapy, CRTAM, DNA-PK

## Abstract

Understanding how tumor cells interact with tumor-infiltrating lymphocytes (TILs) is crucial for improving immunotherapy, yet protein-level changes remain largely unexplored. To address this, we profile the early responses of patient-derived melanoma cells co-cultured with matched autologous TILs. To distinguish tumor from TIL proteomes without physical sorting, we apply stable isotope labeling by amino acids in cell culture (SILAC) coupled with Orbitrap Astral data-independent acquisition (DIA) mass spectrometry (MS). This approach enables cell type-specific profiling of protein phosphorylation and degradation, alongside bulk analysis of the early newly synthesized proteome during active immune attack. Our analyses resolve interferon-γ-dependent changes in melanoma cells, identify the cytotoxic and regulatory T cell molecule (CRTAM) as a selective marker of reactive TILs, and reveal rapid tumor-intrinsic activation of DNA damage response-associated kinases, exposing potential therapeutic vulnerabilities. Overall, this framework provides a powerful resource for dissecting tumor-immune interactions to guide biomarker discovery and advance immunotherapy.

## Highlights

• SILAC-DIA proteomics maps tumor-TIL signaling without cell separation
• T cell attack drives a faster, synergistic proteomic response than IFN-γ alone
• CRTAM marks a subpopulation of tumor-reactive cytotoxic T cells
• Tumor cells rapidly activate DNA damage kinases upon immune attack

## Graphical abstract

**Figure:**
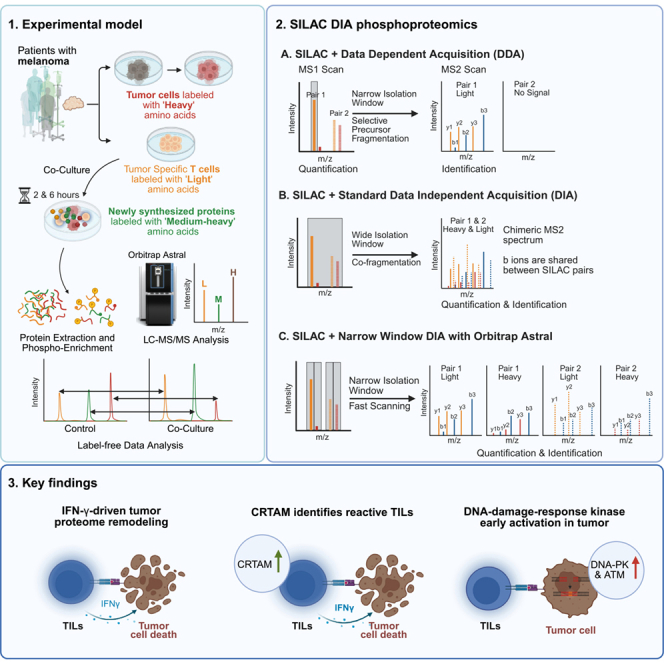

## Teaser

Franciosa et al. map the complex protein signaling between patient-derived melanoma and autologous T cells during active tumor attack. By analyzing these interactions without separating the cells, they uncover new immune reactivity markers and tumor survival mechanisms, providing a valuable resource to improve cancer immunotherapy.

## Introduction

Enhancing a patient’s immune system through immune checkpoint blockade (ICB) can cure patients with metastatic melanoma and other malignancies. However, nearly half of the patients with melanoma do not respond to current ICB therapies^1^^,^^2^ and response rates across other solid tumors remain heterogeneous,^3^ leaving a substantial proportion of patients without durable clinical benefit. Treatment failure has been associated with a low number of immunogenic antigens, defective antigen presentation, and/or the expression of alternative immune checkpoint molecules.^4^ Tumor-infiltrating lymphocytes (TILs) are specialized immune cells capable of targeting cancer cells, and their pre-existing activity within immune “hot” tumors is associated with a greater likelihood of ICB response.^5^ However, no consensus biomarkers currently exist to quantify intratumoral TIL activity and accurately predict ICB efficacy in individual patients. This highlights the urgent need for both improved therapeutic strategies and reliable predictive biomarkers for therapy response.

Previous efforts to identify resistance mechanisms or biomarkers in hot tumors have largely relied on genomics and transcriptomics.^6^^,^^7^^,^^8^ While informative, these approaches fail to capture early and dynamic signaling rewiring events mediated by protein post-translational modifications (PTMs). PTMs, particularly reversible and site-specific phosphorylation catalyzed by protein kinases, act as critical regulators of protein function and cellular signaling.^9^ Mass spectrometry (MS)-based phosphoproteomics, the large-scale study of protein phosphorylation by liquid chromatography-MS,^10^ enables the identification of about 30,000 phosphorylation sites within just half an hour of data acquisition,^11^ providing a functional snapshot of cellular kinase activity.^12^

We previously demonstrated that TILs isolated from melanoma patients remain reactive against autologous tumor cells in 2D co-cultures.^13^ However, applying phosphoproteomics to such co-culture systems presents a key challenge: cell lysis eliminates cell identity information, while manual separation is inefficient.^8^ Fluorescence-activated cell sorting (FACS) could be an alternative, but no published protocols exist for phosphoproteomics of sorted cells.

To address these limitations, we employed a stable isotope labeling by amino acids in cell culture (SILAC)-based strategy^14^ to computationally discriminate tumor cells from TILs without the need for physical separation. To capture signaling dynamics during the interaction, the co-culture was performed in a medium containing a third stable isotopic label, distinct from the initial tumor and TIL labels. While this experimental logic has previously yielded insights into tumor-T cell networks using standard data-dependent acquisition,^15^ we advanced this methodology by coupling it with data-independent acquisition (DIA) using the Orbitrap Astral mass spectrometer.^16^ Although SILAC or other MS1-based multiplexing have been successfully combined with DIA in other contexts,^17^^,^^18^^,^^19^^,^^20^^,^^21^ this specific combination has not previously been applied to distinguish different cell populations. This strategy allowed us to simultaneously analyze newly synthesized proteins alongside cell type-specific protein stability and phosphorylation site changes with unprecedented depth. Finally, we provide an interactive web resource (https://giu-f.github.io/Melanoma_Proteomics/) to enable the community to explore this multidimensional dataset.

## Results

### Loss of phosphorylation dynamics due to prolonged sample processing

To study the early signaling events during T cell-mediated attack of melanoma cells, we used a patient-derived 2D co-culture system in which melanoma cell lines established from tumor biopsies are targeted by autologous TILs isolated from the same biopsies.^13^

**Figure 1:**
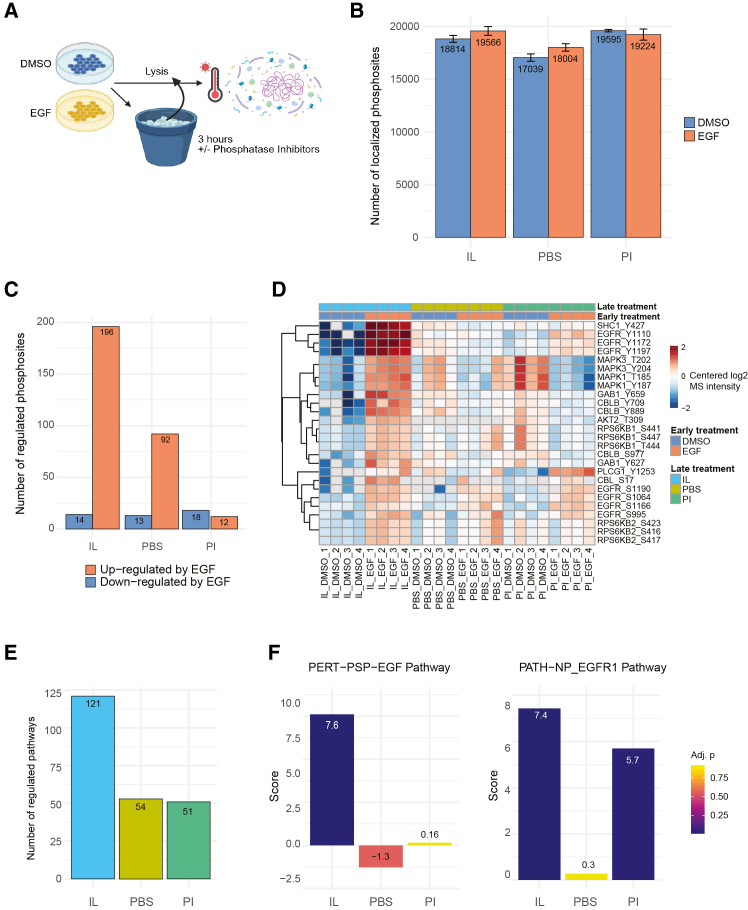
Phosphoproteomic profiling of SCC-25 cells stimulated with EGF and lysed immediately or after incubation on ice (A) Schematic drawing of the phosphoproteomics experiment performed in SCC-25 cells. Following stimulation with EGF for 8 min, SCC-25 were either lysed immediately using a hot lysis buffer (IL) or resuspended in PBS and further incubated on ice for 3 h prior to lysis with (PI) or without (PBS) the addition of phosphatase inhibitors. Created with BioRender. (B) Localized phosphosite identifications per condition. Data are presented as mean ± SD (*n* = 4 biological replicates). (C) Number of up- and down-regulated phosphosites per contrast. (D) Heatmap of mean-centered log2 MS intensities of the significantly regulated phosphosites in at least one contrast belonging to the ErbB KEGG signaling pathway (*n* = 33 phosphosites). Mean centering was performed by condition. (E) Number of significantly regulated (adjusted *p* ≤ 0.05) pathways by PTM-SEA in at least one contrast. (F) PTM-SEA score representing phosphorylation alterations in response to EGF. See also Table S1.

To develop an optimal method for analyzing phosphorylation site changes in this system using MS-based phosphoproteomics, we first evaluated whether FACS could be used to physically separate tumor cells and TILs while preserving their *in vivo* phosphorylation state prior to MS sample preparation. As a model system, we used human SCC-25 squamous cell carcinoma cells stimulated with recombinant epidermal growth factor (EGF) for 8 min. Cells were lysed immediately after stimulation using a hot denaturating SDS-based lysis buffer to preserve phosphorylation by inactivating proteases, kinases, and phosphatases. To simulate FACS-related sample processing, cells were incubated on ice for 3 h prior to lysis, with or without the addition of phosphatase inhibitors (Figure 1A). This experiment showed that while the total number of identified phosphorylation sites was only slightly reduced by the prolonged incubation, this effect was negligible in the presence of phosphatase inhibitors (Figure 1B). However, most of the significantly EGF-regulated phosphosites (Table S1) lost their regulation following incubation on ice, and this effect was surprisingly even more pronounced when phosphatase inhibitors were added (Figure 1C). When focusing on the EGFR signaling pathway, we found that the dynamic regulation of key phosphosites involved in the pathway was severely compromised by prolonged incubation on ice (Figure 1D). Phosphatase inhibitors did not rescue this loss of regulation and, in some cases, even led to an inverse regulation. When performing PTM-signature enrichment analysis (PTM-SEA), prolonged sample handling markedly reduced the number of significantly regulated pathways (Figure 1E; Table S1), with EGFR signaling entirely abrogated in PBS-treated samples and only partially retained in the presence of phosphatase inhibitors (Figure 1F).

These findings suggest that prolonged processing steps such as FACS sorting interfere with the detection of dynamic phosphorylation changes. Therefore, they are not compatible with phosphoproteomics methods aimed at capturing signaling responses.

### Validation of a multiplexed SILAC-DIA workflow for cell type-resolved proteome profiling

**Figure 2:**
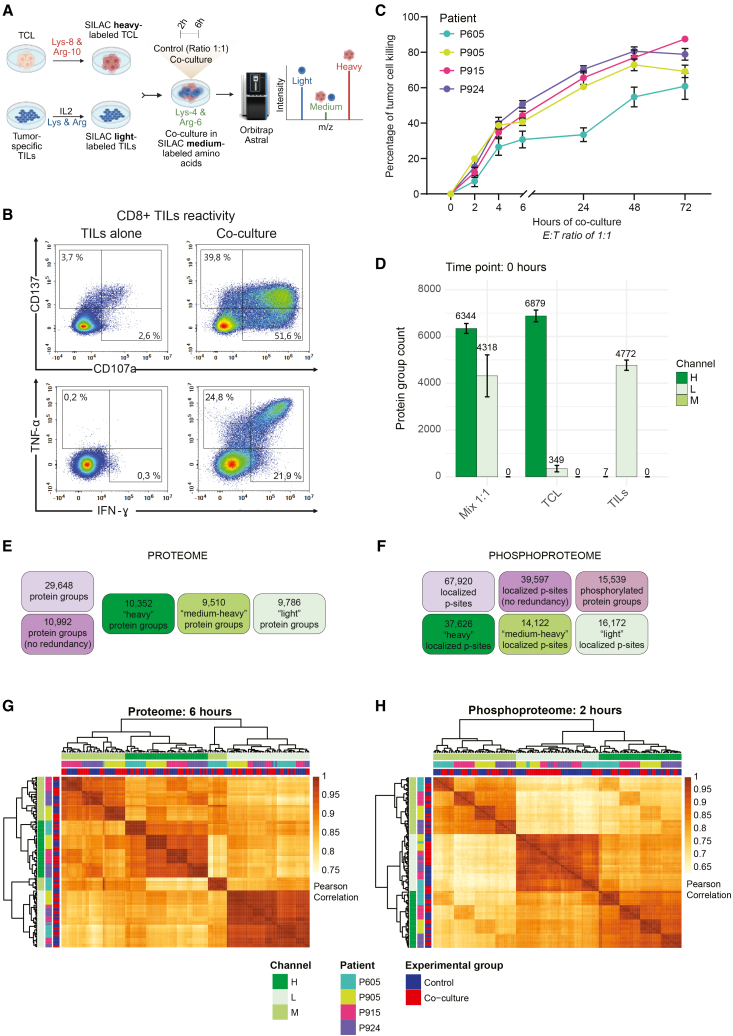
SILAC-DIA proteomic and phosphoproteomic analysis of patient-derived melanoma cells under attack by autologous TILs (A) Schematic drawing of the SILAC-DIA-based proteomics experiment performed in one patient pair. Created with BioRender. (B) Representative density plots of flow cytometric analysis of surface expression of CD137 and CD107a, and intracellular expression of TNF-α and IFN-γ in activated CD8^+^ TILs from patient 905. TILs were co-cultured for 8 h with autologous melanoma cells at effector:target (E:T) ratio 3:1. (C) Autologous TIL-mediated killing of melanoma cells from four different patients by xCELLigence real-time cell analysis at an E:T ratio of 1:1. Data are presented as mean ± SD (*n* = 3–4 technical replicates). (D) Protein group identifications in the proteomics pilot experiment performed in patient 905 at time point 0 h (before co-culture). Data are presented as mean ± SD (*n* = 4 biological replicates). Samples were analyzed on 180-sample-per-day (SPD) gradient. (E and F) Number of identifications in the four patients’ proteomics and phosphoproteomics experiments, respectively. Samples were analyzed on a 30-SPD gradient. (G and H) Pearson correlation heatmaps of log2 MS intensities, including all 96 samples analyzed in the four patients’ proteomics (6 h) and phosphoproteomics (2 h) experiment, respectively. See also Figures S1 and S2; Table S2.

Because FACS could not be used, cell type-specific information had to be obtained without physically separating tumor cells and TILs prior to lysis. To achieve this, we employed SILAC labeling of tumor cells using “heavy”-labeled amino acids (K8 and R10), while TILs were left unlabeled (“light”: K0 and R0). The two cell types were co-cultured for 2 and 6 h, respectively, in media containing “medium-heavy”-labeled amino acids (K4 and R6). This approach ensures that newly synthesized proteins, which cannot be assigned to a specific cell type, can be categorized accordingly. As a control, tumor cells and TILs were incubated separately and mixed immediately before lysis (Figure 2A).

The tumor-TIL pairs (Table S2) were selected based on high anti-tumor reactivity and efficient tumor cell killing in preliminary assays. Flow cytometric assessment of anti-tumor reactivity confirmed a robust TIL activation across all selected pairs, demonstrated by the up-regulation of the costimulatory molecule CD137,^22^ the degranulation marker CD107a,^23^ and the increased secretion of IFNγ and TNFα (Figures 2B and S1A). To capture early signaling while maintaining comparable cytotoxicity without excessive cell death, effector-to-target (E:T) ratio titrations were performed (Figure S1B), leading us to select a 1:1 E:T ratio for all subsequent MS experiments (Figure 2C). Prior to performing any experiment, melanoma cell lines were analyzed by flow cytometry to confirm minimal fibroblast contamination (Figure S1C). Additionally, TILs were characterized for the relative abundance of CD8^+^ and CD4^+^ T cells, highlighting that three of the four TIL products (P905, P915, and P924) were predominantly CD8^+^ T cells, whereas one (P605) contained approximately equal proportions of CD8^+^ and CD4^+^ T cells (Figure S1D).

To test the quantitative performance of the SILAC strategy, we selected one patient pair to perform a pilot experiment. Full-proteome samples were analyzed on the Orbitrap Astral mass spectrometer using narrow-window DIA (nDIA),^16^ and the resulting MS data were processed with DIA-NN 2.0^24^ using the plexDIA module.^20^ Tumor cells and TILs were also collected and analyzed separately at time point 0 h, before adding the medium-heavy amino acids. TIL-only samples yielded almost exclusively light-labeled peptide identifications, with a calculated mean false discovery rate (FDR) in the heavy channel of 0.1%. Slightly more light-labeled peptides were detected in tumor samples, likely due to a not-full incorporation of heavy amino acids. Importantly, no medium-heavy-labeled peptides were detected in either cell type at 0 h prior to co-culture, yielding a calculated mean FDR in the medium-heavy channel of 0% (Figure 1D).

Using the SILAC-based co-culture approach, we next asked whether tumor cells and TILs could simply be separated after co-culture to obtain clean, cell type-specific proteomes. If this were feasible, SILAC labeling would not be required. However, manual separation resulted in substantial cross-contamination, with light peptides detected in tumor cell fractions, and vice versa, already evident at the 2-h time point (Figure S2A).

Quantification in DIA-NN 2.0 leverages both MS1 and MS2 data to enable precursor quantification.^25^ To evaluate whether the MS2-oriented nDIA method (method 1)^16^ was the most optimal for multiplexed DIA in this context, we compared it to an MS1-oriented nDIA method (method 2). Given that TILs are smaller and contribute less protein than tumor cells in the mixed tumor-TIL sample, we also tested whether a wider DIA window (method 3) would improve ion accumulation and detection depth for TIL-derived peptides. Among the three, method 1 outperformed the others, yielding the highest number of identifications (Figure S2B) and the lowest coefficients of variation (Figure S2C), serving as a proxy for quantification precision.

To further validate the reliability of the phosphosite localization algorithm, we performed a phospho-alanine decoy search on phospho-enriched light tumor samples. This analysis demonstrated high localization precision, with alanine decoy identifications remaining negligible as the localization probability threshold increased (Figure S2D). At a standard class I localization threshold (>0.75), the empirical false localization rate was 1.3%, which dropped to a highly stringent 0.3% at a threshold of 0.99 (Figure S2E). Crucially, the empirical FDR in the empty medium and heavy channels remained at 0% across all localization thresholds, indicating no detectable cross-channel interference (Figure S2D). Altogether, these metrics confirmed the effectiveness of the site-localization algorithm and the quantitative robustness of the plexDIA module for our experimental setup.

### Deep proteomic and phosphoproteomic analysis of melanoma-TIL pairs

To comprehensively investigate the signaling rewiring underlying the interaction between patient-derived melanoma cells and TILs, we performed a large co-culture experiment using cells from all four patients for both proteome and phosphoproteome analyses. Each condition was assessed in six biological replicates at two time points (2 and 6 h). The heavy-SILAC labeling efficiency was higher than 90% in all cell lines. Light channel FDR ranged from 0% to 0.4%, while medium-heavy channel FDR was 0% across all patients (data not shown).

Across all samples, we confidently identified approximately 30,000 protein groups in the proteome data. Of these, 10,352 were detected in the heavy channel, 9,786 in the light channel, and 9,510 in the medium-heavy channel. Approximately 11,000 protein groups were identified regardless of channel assignment (Figure 2E); 10,175 were shared between at least two channels and 8,481 shared across all three (Figure S2F).

Due to the inherent size differences between melanoma and T cells, the light channel (TILs) exhibited the highest proportion of missing values. On average, 8,618 and 8,048 protein groups were identified per run in the heavy channel at 2 and 6 h, respectively, while the light channel yielded an average per run of 4,151 and 3,715 identifications at the same time points. In the medium-heavy channel, which captures newly synthesized proteins, the number of identifications was initially low (2,645 per run at 2 h), consistent with the detection of fast-turnover proteins only. However, by 6 h, the average number of identifications rose to 6,211 per run on average, indicating that a substantial portion of the proteome had undergone synthesis and was incorporated into the medium-heavy label by this time (Figure S2G).

Given the lowest rate of protein turnover, we selected the 2-h time point for phosphoproteomic analysis to focus on cell type-specific signaling in pre-existing (“old”) proteins, minimizing interference from the few newly synthesized proteins labeled in the medium-heavy channel. Samples were processed through low-input phosphoproteomics.^26^ Across all samples, we confidently identified approximately 70,000 localized phosphorylation sites on 15,539 protein groups. Of these phosphosites, 37,626 were detected in the heavy channel, 16,172 in the light channel, and 14,122 in the medium-heavy channel. Approximately 40,000 phosphorylation sites were identified regardless of channel assignment (Figure 2F); 19,290 were shared between at least two channels and 9,033 were shared across all three (Figure S2H). On average, 16,157 and 8,048 phosphorylation sites were identified per run in the heavy channel, while the light channel yielded an average of 5,037 identifications and the medium-heavy channel yielded 3,892 (Figure S2I).

We generated Pearson correlation heatmaps for the proteome data at 6 h (Figure 2G) and for the phosphoproteome data at 2 h (Figure 2H). In all heatmaps, the main clustering was driven by the SILAC channel, confirming that our multiplexing approach effectively distinguishes both the cellular origin (tumor cells vs. TILs) and the fraction of newly synthesized proteins. At 2 h, the medium-heavy channel clustered separately from the heavy and light channels, whereas at 6 h, it clustered together with the heavy channel, suggesting that the majority of newly synthesized proteins at later time points originate from tumor cells (which are larger and thus contain more protein). The only exception was samples from patient 605 whose medium-heavy channel was clustered with the light channel, suggesting that T cells are producing more proteins in this system. This deviation aligns with the highest prevalence of CD4-positive TILs in this patient (Figure S1D), which sustain higher intrinsic translational rates compared to the frequently exhausted and metabolically restricted CD8^+^ TILs.^27^^,^^28^^,^^29^ Within the heavy and medium-heavy channels, samples were always clustered by patient, with three patients (P905, P915, and P924) clustering separately from patient P605. Interestingly, TILs did not show a marked patient-specific clustering. We observed a certain level of clustering by condition (control vs. co-culture) in the heavy and light channels for the phosphoproteome and in the medium-heavy channel for the proteome. This separation improved when we used Euclidean distance, which allows for missing values, as the clustering metric (Figure S2J).

### Proteome changes upon T cell attack

On the proteome data, we performed differential expression analysis to identify proteins significantly regulated upon T cell attack across all patients (Table S3). Proteins showing regulation in the heavy or light channels reflect cell type-specific changes and are primarily interpreted as differentially degraded upon co-culture, although a minor contribution from newly synthesized proteins incorporating recycled amino acids cannot be excluded. In contrast, proteins regulated in the medium-heavy channel represent changes in protein synthesis upon co-culture and may originate from either tumor cells or TILs. Most of the protein abundance changes were observed in the medium-heavy channel at 6 h, with 263 up-regulated proteins and 80 down-regulated.

**Figure 3:**
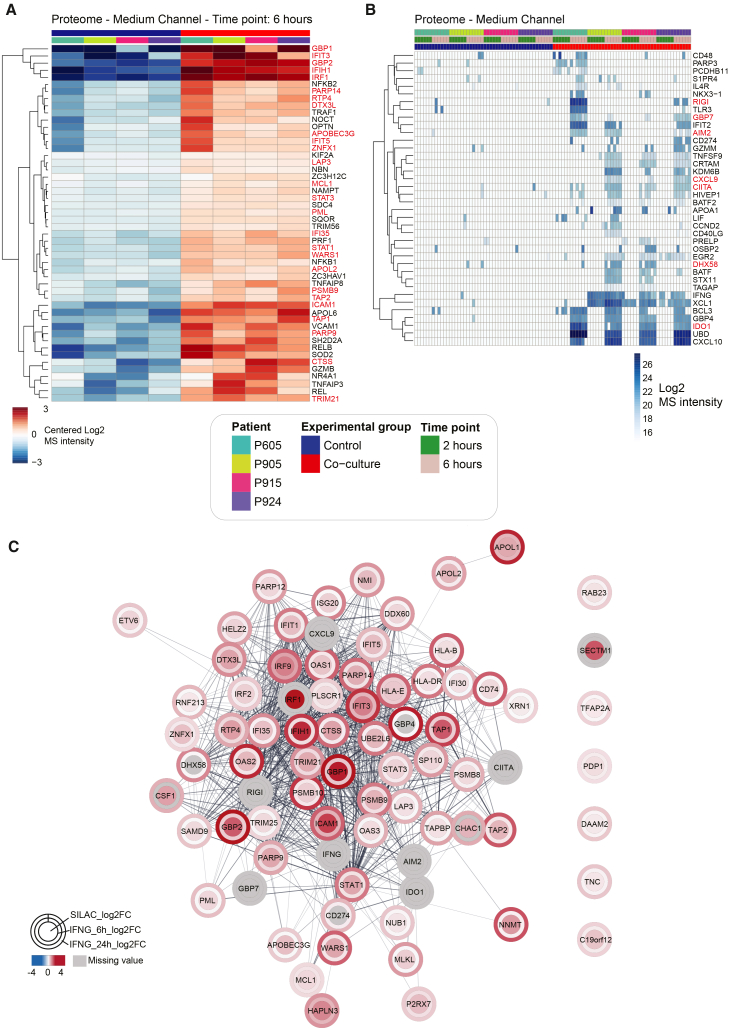
Global changes in the newly synthesized proteome (medium-heavy channel) upon T cell attack and comparison with IFN-γ stimulation (A) Heatmap of mean-centered log2 MS intensities of the 50 up-regulated proteins with the lowest adjusted *p* value in the medium-heavy channel at 6 h. Missing values were imputed with the minimum value of the filtered matrix before mean centering, which was performed by patient. Proteins labeled in red are induced by IFN-γ (therefore, also displayed in C). (B) Heatmap of log2 MS intensities of the 38 proteins preferentially expressed upon co-culture. Missing values are colored in white. Proteins labeled in red are induced by IFN-γ (therefore, also displayed in C). (C) Functional STRING protein network of the proteins up-regulated both by T cell attack in the medium-heavy channel at 6 h and by IFN-γ stimulation of melanoma cells either at 6 or 24 h. Proteins without a calculated log2 FC are exclusively present in one condition. See also Figure S3; Tables S3 and S4.

Proteins showing up-regulation in the medium-heavy channel showed a high degree of similarity across the four different patients (Figure 3A). Since many immune-related proteins may exhibit a high number of missing values in the control condition of the medium-heavy channel, we systematically searched for proteins with such a pattern and identified 38 proteins preferentially expressed upon co-culture (Figure 3B) and 78 preferentially not expressed (Table S3). Among proteins up-regulated or exclusively induced upon T cell attack, we identified the adhesion molecules ICAM1 and VCAM1 that may facilitate stable immune synapse formation, enabling cytotoxic T lymphocyte (CTL)-mediated killing, as supported by the presence of PRF1 and GZMB, key components of the CTL arsenal. We also found several known interferon-γ (IFN-γ) targets, for instance, STAT1 and IRF1. At the same time, immune checkpoint proteins CD274 (PD-L1) and IDO1 emerged, suggesting attempts by tumor cells to resist immune attack. Activation of both canonical (NFKB1) and non-canonical (NFKB2 and RELB) NF-κB pathways, along with regulators like TNFAIP3, reflected sustained inflammatory signaling. Innate immune sensors including RIG-I (DDX58), DHX58, TLR3, and AIM2 were also up-regulated, pointing to nucleic acid sensing triggered by immune-mediated stress. Finally, proteins such as NAMPT, SOD2, and OPTN suggested metabolic and oxidative adaptations that help tumor cells cope with the inflammatory microenvironment.

### Deconvolution of the interferon-γ-dependent proteome changes upon T cell attack

We have previously shown that T cell attack induces broader transcriptional changes in tumors compared to IFN-γ in melanoma.^8^ To confirm this on the proteome level, we stimulated three different patient-derived tumor cell lines (P905, P915, and P924) with recombinant IFN-γ for 6 and 24 h for MS-based proteome analysis. In total, we identified 8,693 protein groups. Of these, approximately 8,400 were identified per cell line, 8,017 were shared between cell lines, and a mean of approximately 7,300 protein groups was identified per run. We performed differential expression analysis to identify proteins significantly regulated by IFN-γ across all patients (Table S4). IFN-γ treatment up-regulated 26 and 125 proteins at 6 and 24 h, respectively, while down-regulating 4 and 119 proteins at the same time points. These expression patterns were highly consistent across patients and were most distinct after 24 h of IFN-γ exposure (Figure S3A). Furthermore, we identified 18 proteins unique to the IFN-γ-treated samples and 12 unique to the controls (Table S4).

When we compared newly synthesized proteins upon T cell attack with those affected by IFN-γ treatment (Figure S3B; Table S4), we observed minimal overlap among down-regulated proteins, whereas up-regulated proteins were largely shared between the two conditions (Figure 3C). Nearly half of the proteins up-regulated by IFN-γ at 24 h were also induced after just 6 h of T cell attack, suggesting a synergistic response likely driven by the combined action of IFN-γ and other cytokines released during co-culture, as well as the activation of additional signaling pathways. This observation was supported by a lack of correlation between fold changes in the co-culture and those induced by IFN-γ at 6 h (R^2^ = 0.004; Figure S3C), contrasting with the stronger positive correlation observed at 24 h (R^2^ = 0.2; Figure S3D).

### Linking proteomic responses to functional outcomes of T cell attack

**Figure 4:**
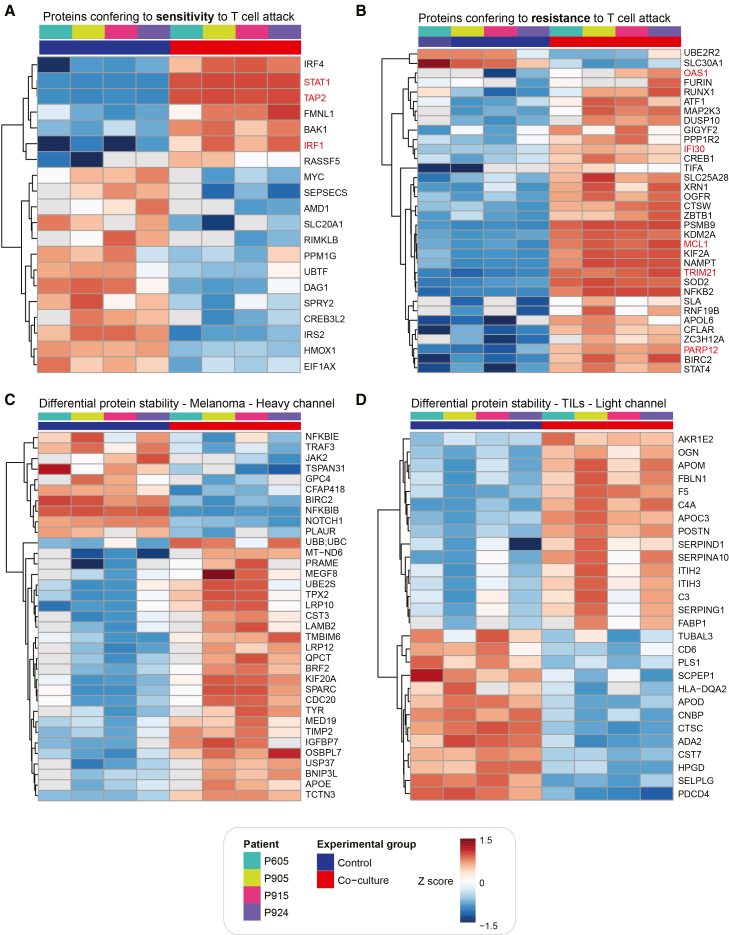
Impact of T cell attack on functional drivers of susceptibility and on cell type-resolved protein stability (A and B) Heatmaps of *Z*-scored medium channel log2 MS intensities for proteins conferring sensitivity (A) or resistance (B) to T cell attack. (C and D) Heatmaps of *Z*-scored log2 MS intensities of proteins significantly regulated in the heavy (C) or light (D) channel and not showing the same trend in the medium-heavy channel. In all heatmaps, *Z* score was performed by patient and channel. See also Table S3.

Whole-genome CRISPR-Cas9 screens have been widely used to dissect T cell-tumor interactions and identify genes that influence tumor cell survival during immune attack.^30^ In this study, we employed *in vitro* CRISPR screen data from two melanoma models: murine B16 cells engineered to express the model antigen ovalbumin (Ova)^31^ and human D10 cells that endogenously express the tumor-associated antigen MART-1.^32^ In both systems, tumor cells were challenged with antigen-specific CTLs. Genes significantly enriched (positive selection) at the endpoint of the screen are those whose knockout improves cell survival under T cell pressure, while significantly depleted genes (negative selection) are those whose loss reduces tumor viability under T cell pressure. To integrate these findings with protein-level responses, we combined CRISPR screen results with our proteomic data from tumor cells exposed to T cell attack. Based on the direction of the change in both datasets, we defined two groups of proteins: one where genes were either enriched and up-regulated or depleted and down-regulated, suggesting a potential role in promoting sensitivity to T cell killing (Figure 4A), and another where genes were enriched and down-regulated or depleted and up-regulated, indicating a possible contribution to resistance (Figure 4B). Among the up-regulated proteins conferring sensitivity, we identified the IFN-γ targets STAT1, IRF1, and TAP2. Notably, IFN-γ signature was previously associated with a high correlation to ICB therapy,^33^ while TAP2 down-regulation has been shown to drive immune evasion and immunotherapy resistance.^34^ In this group, however, some IFN-γ targets (MCL1, PARP12, IFI30, TRIM21, and OAS1) showed the opposite trend, being present in the resistance group, indicating they may contribute to the protumorigenic role of IFN-γ signaling, promoting immune evasion.^35^ In the resistance group, we also identified the up-regulation of BIRC2 and KDM2A, whose inhibition has been previously associated with increased sensitivity to T cell killing.^36^^,^^37^

### Cell type-resolved analysis of protein stability upon T cell attack

To isolate proteins subject to differential degradation induced by the co-culture environment, we compared the co-culture samples to the control, where tumor cells and TILs were incubated separately and mixed immediately prior to lysis, both for the heavy and light channels. This analysis captures a snapshot of relative protein stability upon co-culture. To ensure these changes reflected specific degradation/stabilization events rather than protein synthesis artifacts, such as the re-incorporation of recycled labeled amino acids, we excluded proteins that displayed parallel regulatory trends in both the newly synthesized (medium-heavy) and pre-existing (heavy/light) pools. Using this filter, we identified several immune-related proteins differentially degraded both in tumor cells (Figure 4C)—including JAK2 and TRAF3, as well as tyrosinase (TYR)—and in TILs (Figure 4D), such as CD6 and CTSC.

### CRTAM: A novel reactive cytotoxic T lymphocytes activation marker

**Figure 5:**
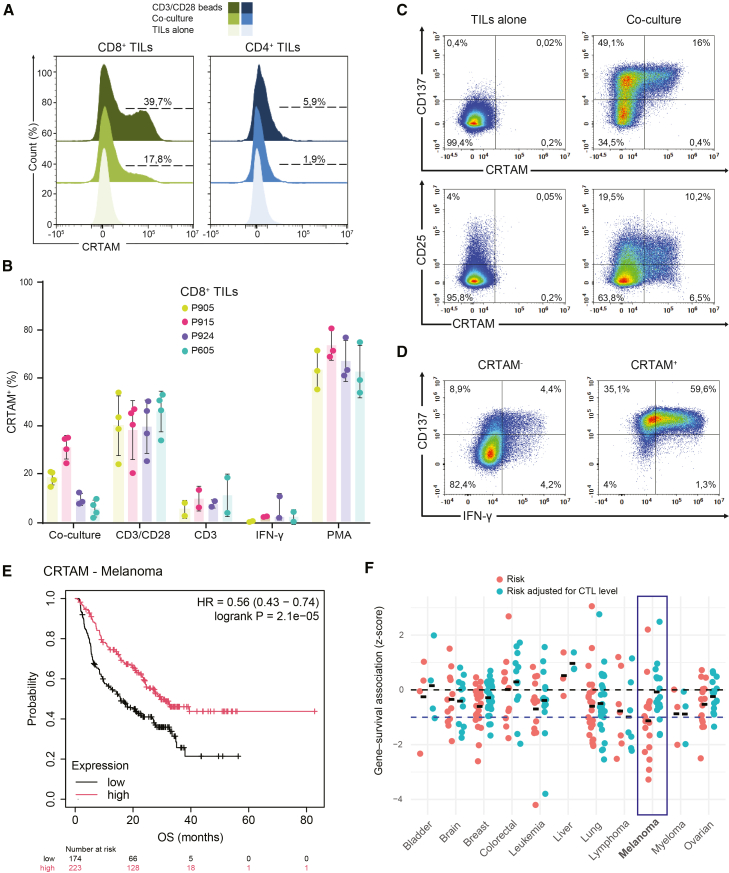
Functional role of CRTAM in melanoma immune recognition (A) CRTAM surface expression on CD4^+^ and CD8^+^ TILs from patient 905, assessed by flow cytometry after 6 h of stimulation with CD3/CD28 beads or co-culture with autologous melanoma cells. (B) Percentage of CRTAM in CD8^+^ TILs from all four patients after 6 h of co-culture with autologous melanoma cells or stimulation with anti-CD3 antibody, CD3/CD28 beads, IFN-γ, and PMA/ionomycin. Data are presented as mean ± SD (*n* = 2–4 biological replicates, each with three technical replicates). (C) Representative density plots of flow cytometric analysis showing surface co-expression of CRTAM with CD137 or CD25 on CD8^+^ TILs from patient 905 after 6 h of co-culture with autologous melanoma cells. (D) Intracellular expression by flow cytometry of CD137 and IFN-γ in CRTAM^+^ and CRTAM^−^ CD8^+^ TILs from patient 915 after 8 h of co-culture with autologous melanoma cells. Experiments in (A)–(D) were performed at an E:T ratio of 1:1. (E) Kaplan-Meier (KM) curve showing the association between CRTAM gene expression and survival in a cohort of 423 melanoma patients undergoing treatment with ICB. (F) Analysis of the Tumor Immune Dysfunction and Exclusion (TIDE) database showing the *Z* score of the effect of CRTAM expression on overall survival across multiple cancer types in a CoxPH model, before and after adjusting for CTL cell infiltration. A negative *Z* score means that higher CRTAM expression is associated with lower death risk. See also Figures S4 and S5.

To demonstrate that our data capture biologically meaningful changes, we functionally validated the role of the transmembrane protein cytotoxic and regulatory T cell molecule (CRTAM, also known as CD355), which was uniquely detected in the medium-heavy channel of the proteomics data after 6 h of co-culture in three of four patients (Figure 3B). Flow cytometry analysis across all four patients confirmed CRTAM up-regulation almost exclusively on CTLs following tumor engagement (Figure 5A and S4A-B), with P905 and P915 showing more than 15% CRTAM^+^ CD8^+^ TILs (Figure S4A). This up-regulation required CTL activation through the TCR/CD3 complex, was further enhanced by CD28 co-stimulation, and was independent of IFN-γ (Figures 5A and 5B). CRTAM expression was rapidly induced upon co-culture, reaching a maximum level between 7 and 24 h before returning to baseline by 48 h (Figure S4C), making CRTAM an early CTL activation marker. CRTAM-positive CTLs co-expressed the reactivity marker TNFRSF9/CD137 and the survival marker IL2RA/CD25, the alpha chain of the IL2 receptor (Figure 5C and S4D), and showed increased secretion of IFN-γ compared to CRTAM-negative CTLs (Figure 5D and S4E). Upon bead stimulation of healthy donor peripheral blood mononuclear cells (PBMCs), CRTAM-positive CTLs demonstrated enhanced proliferative capacity relative to CRTAM-negative CTLs (Figure S4F). These data suggest that CRTAM identifies a subset of tumor-infiltrating CTLs with enhanced anti-tumor activity. In a single-cell RNA sequencing dataset from 33 melanoma tumors,^38^ CRTAM expression was confirmed predominantly on CTLs (Figure S5A); both TNFRSF9/CD137 and IFN-γ transcripts were significantly enriched in CRTAM-high CTLs (Figures S5B–S5E), while IL2RG/CD132, the gamma chain of the IL2 receptor, was enriched in CRTAM-positive cells (Figure S5F).

To assess the clinical relevance of CRTAM in CTL-mediated melanoma killing, we analyzed the correlation between CRTAM gene expression and overall survival (OS) in a clinical cohort of 423 patients with melanoma undergoing treatment with ICB^39^ and observed that high CRTAM expression predicted improved OS (Figure 5E), suggesting that it may serve as a surrogate marker of anti-tumor immune activity and clinical benefit. To assess whether the association between CRTAM and improved OS extended beyond this cohort and melanoma, we evaluated the prognostic effect of CRTAM expression across multiple melanoma cohorts and cancer types using a Cox proportional hazards (CoxPH) regression model implemented in the TIDE (Tumor Immune Dysfunction and Exclusion) algorithm.^40^^,^^41^ High CRTAM expression was associated with reduced risk of death in most cancer types, with melanoma showing the strongest and most consistent protective effect (Figure 5F). Adjusting for CTL infiltration markedly reduced the predictive value of CRTAM in melanoma, indicating that its association with survival was largely mediated by CTL abundance. Consistent with this, CRTAM expression positively correlated with CTL levels across cohorts, most strongly in melanoma, close to CD8A itself (Figure S5G). Together, these findings suggest that CRTAM expression defines a subset of CTLs whose presence is associated with favorable clinical outcome, consistent with a role in effective anti-tumor immunity.

### Cell type-resolved analysis of protein phosphorylation upon T cell attack

On the phosphoproteome data, since peptides containing medium-heavy-labeled amino acids could originate from either tumor cells or TILs, we focused our analysis on the heavy (tumor-derived) and light (TIL-derived) channels. In total, we identified 1,145 up-regulated phosphosites by T cell attack and 153 down-regulated in the heavy channel, and 743 up-regulated and 128 down-regulated phosphosites in the light channel. Furthermore, we identified phosphosites exclusive to specific conditions, finding 320 and 187 phosphosites unique to the co-culture samples in the heavy and light channels, respectively (Table S5).

**Figure 6:**
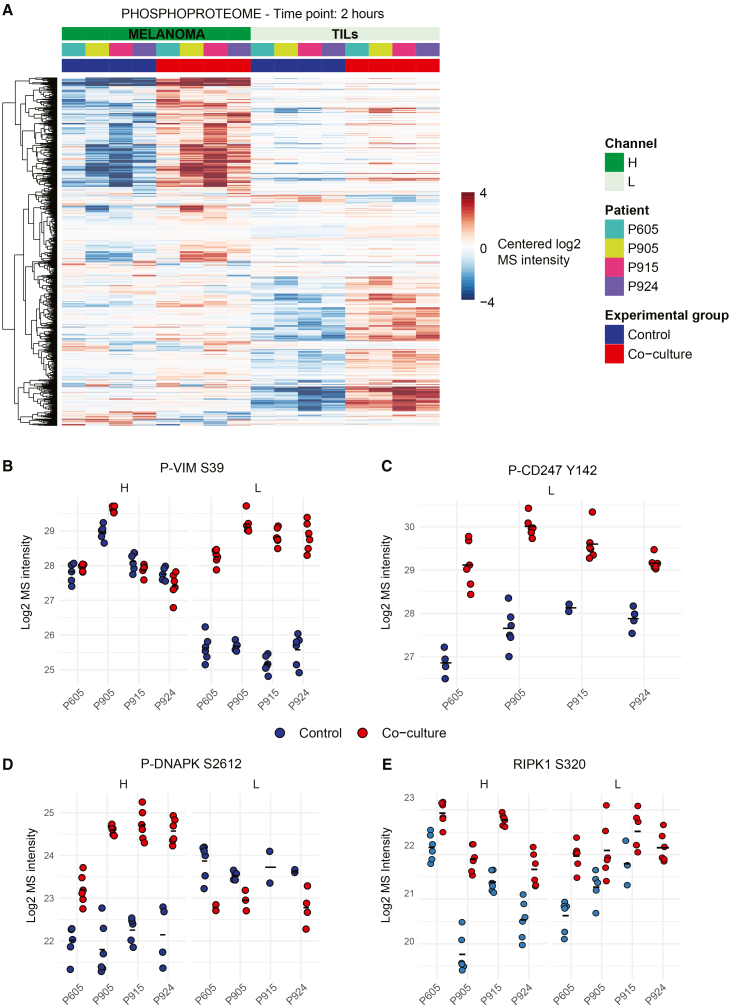
Cell type-resolved analysis of protein phosphorylation upon T cell attack (A) Heatmap of mean-centered log2 MS intensities of the 2,169 significantly regulated phosphosites in the heavy and light channels. Missing values were imputed with the minimum value of the channel-specific filtered matrix before mean centering, which was performed by patient and channel. Phosphosites uniquely present in one channel were imputed with 0 s after mean centering. Rows were clustered with the maximum metric distance. (B–E) Log2 MS intensities of selected phosphosites. The black dash represents the mean. See also Table S5.

Phosphorylation patterns were profoundly different between melanoma cells and TILs (Figure 6A). Among phosphosites with an associated function in the PhosphoSitePlus database^42^ (Table S5) and identified in both cell types, the site most preferentially up-regulated in TILs was serine 39 on vimentin (Figure 6B), an AKT1 target known to affect cell motility and cytoskeleton reorganization. Among the most regulated functional phosphosites in TILs but not identified in melanoma cells, we found several T cell activation markers, for example, tyrosine 142 on CD3ζ (Figure 6C), a crucial component of the T cell receptor (TCR) signaling complex. The most regulated functional phosphosite in melanoma cells, also identified in TILs, was serine 2612 on DNA-PK (Figure 6D), known to enhance the process of DNA repair. Among the sites commonly regulated in both cell types, we identified serine 320 on RIPK1 (Figure 6E), a target of MAPKAPK2 (MK2) and TAK1 kinases in response to inflammatory stimuli such as TNF-α. Phosphorylation at this site inhibits RIPK1-mediated apoptosis by preventing its interaction with FADD and caspase-8.^43^^,^^44^^,^^45^^,^^46^

Using pathway enrichment analysis^47^ (Figure S6A; Table S6), in melanoma cells we observed significant enrichment of DNA damage response (DDR) pathways under T cell attack, including those mediated by ATM and ATR signaling. Interestingly, melanoma cells also exhibited enrichment in innate immune signaling pathways typically associated with antiviral and antibacterial responses (RIG-I-like receptor signaling pathway), suggesting that the intracellular signaling landscape of tumor cells under immune attack mirrors the response of immune cells to pathogenic insults. In contrast, several pathways involved in cell motility were significantly down-regulated upon attack, potentially reflecting a shift from a migratory to a defensive state. Conversely, in TILs, we observed significant enrichment of pathways related to cell motility and cytoskeletal remodeling upon engagement with melanoma cells, consistent with active migration and immune synapse formation. Additionally, pathways associated with TCR activation were strongly enriched, confirming that TILs were functionally responding to tumor antigens during the interaction. Very few pathways showed a shared regulation between the two cell types. This was confirmed by a statistically significant negative linear association between the two cell types at pathway level (intercept = −0.16, *p* = 0.007), with the model explaining only a small fraction of the variance (R^2^ = 0.03), indicating that the overall pathway patterns are mostly unrelated in the two cell types (Figure S6B).

**Figure 7:**
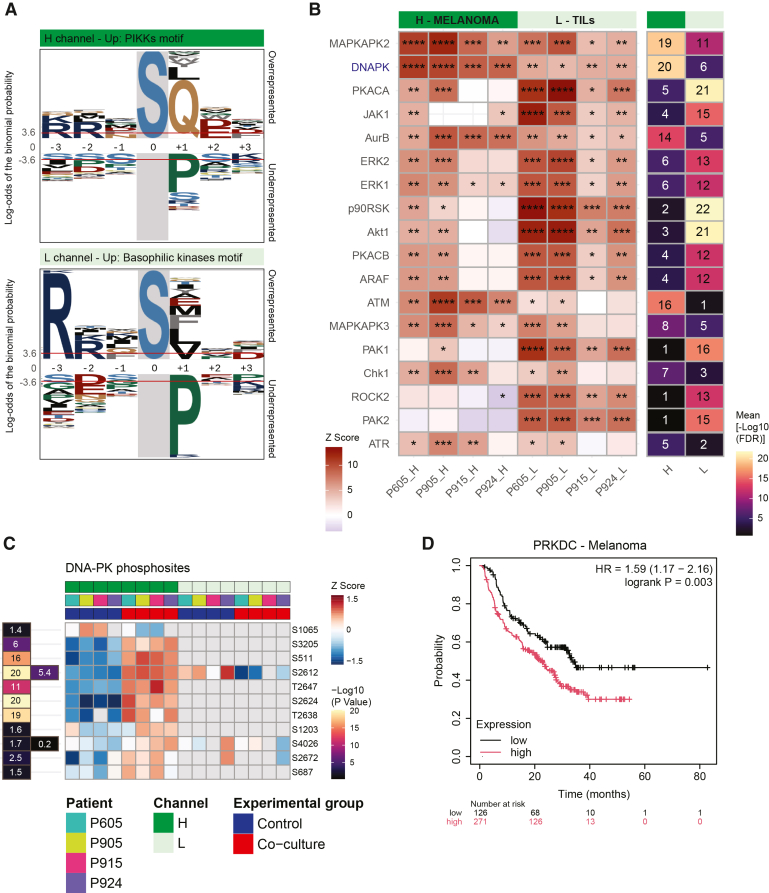
Cell type-resolved analysis of kinase signaling upon T cell attack (A) Amino acid motif over-represented among up-regulated phosphosites in each channel. (B) Heatmaps of RoKAI *Z* scores (left) and the mean of –log10(FDR) across the four patients (right). The 10 kinases with the highest sum of –log10(FDR) across the four patients per channel were selected for this plot, for a total of 18 unique kinases. Significance is represented by asterisks: ∗FDR ≤ 0.05; ∗∗DR ≤ 1e−5; ∗∗∗FDR ≤ 1e−10; ∗∗∗∗FDR ≤ 1e−20. (C) Heatmaps of *Z*-scored log2 MS intensities (right) and –log10(*p* value) (left) for PRKDC phosphosites with a *p* value ≤ 0.05. (D) KM curve showing the association between PRKDC gene expression and survival in a cohort of 423 melanoma patients undergoing treatment with ICB. See also Figures S6 and S7; Table S6.

In melanoma cells, motif enrichment analysis^48^ highlighted the over-representation of a glutamine residue at the +1 position upon attack, which represents the sequence motif for the phosphatidylinositol 3-kinase-related kinases (PIKKs): ATM, ATR, and DNA-PK^49^^,^^50^ (Figure 7A). Conversely, In TILs, the basophilic kinase motif R/K-R/K-x-pS/pT was over-represented upon interaction with melanoma cells, highlighting major differences in kinase activation between the two cell types.

Next, we employed the RoKAI algorithm to assess kinase activity upon T cell attack, by analyzing alterations in the phosphorylation of known kinase substrates and their functional network^51^ (Figure 7B; Table S6). Confirming earlier analyses, attacked melanoma cells preferentially enriched kinases central to the DNA damage response (DDR). These included DNA-PK and ATM, which repair double-strand breaks,^52^ alongside the replication stress regulators ATR and Chk1.^53^ The cell cycle kinase Aurora Kinase B was also enriched, as well as MAPKAPK2 and MAPKAPK3, which are p38 MAPK-regulated kinases involved in stress, inflammation, and the DDR.^54^

Multiple kinases enriched in TILs were basophilic (including PKACA, PKACB, p90RSK, Akt1, PAK1/2, and ROCK2), confirming the motif analysis. Moreover, we identified key members of the canonical MAPK signaling pathway, including the proline-directed ERK/2 kinases and their basophilic downstream target p90RSK. The increased PAK1/2 activity aligns with previous findings by Stecker’s lab,^15^ while the enrichment of ROCK2, a regulator of cytoskeleton and cell motility, explains the enrichment of cytoskeleton-related pathways in TILs (Figure S6A).

To confirm the observed kinase activation pattern, we analyzed kinase activity using PTM-SEA.^47^^,^^55^ This analysis confirmed the preferential enrichment of MAPKAPK2, AURKB, MAPKAPK3, Chk1, and ATM in melanoma cells (Figure S7A; Table S6). Additionally, it showed preferential activation of all four p38 MAP kinases (MAPK11, 12, 13, and 14), which are upstream of MAPKAPK2 and MAPKAPK3. This analysis also confirmed preferential enrichment in TILs of Akt, p90RSK, PKACA, and PAK1.

All in all, these analyses confirmed the ability of our approach to resolve phosphorylation-specific signaling events with cell-type resolution.

### DNA-PK: A potential immune resistance kinase in melanoma

To further dissect the identified DNA repair response, we zoomed in on specific effector kinases. This revealed a prominent activation of the DDR machinery, most notably DNA-PK. In melanoma cells exposed to TILs, we observed the up-regulation of multiple DNA-PK phosphorylation sites known to facilitate DNA repair (Figures 7C and S7B).

To investigate the functional relevance of DNA-PK during T cell attack, we first analyzed the association between DNA-PK gene expression and OS in a clinical cohort of patients treated with ICB^39^ and found that high DNA-PK expression predicted worse OS (Figure 7D). We confirmed this negative prognostic association across multiple cancer types (Figure S7C). Importantly, the predictive value of DNA-PK remained after adjusting for the level of T cell infiltration. In line with this, DNA-PK gene expression negatively correlated only weakly with CTL infiltration (Figure S7D), suggesting that DNA-PK expression influences survival independently of CTL cell abundance in the tumor microenvironment (TME).

To further dissect the role of DNA-PK in tumor immune evasion, we examined data from the negative CRISPR screen by Zhang et al.^32^ for DNA-PK, ATM, and key immune checkpoints (PD-L1, PD-L2, TIM-3, and IDO1). None of these genes showed strong negative selection, meaning that their knockout did not significantly impair melanoma cell survival (Figure S7E). However, while DNA-PK knockout did not reach high statistical significance, it showed a negative rank close to the best-performing immune checkpoints, highlighting its potential role as an immune resistance factor *in vivo*, suggesting the role of this kinase in melanoma immune escape.

## Discussion

In this study, we applied a triple SILAC-DIA phosphoproteomics workflow on the Orbitrap Astral MS to resolve early, cell type-specific signaling between patient-derived melanoma cells and autologous TILs. In doing so, we leveraged the established capacity of SILAC to maintain cell identity upon lysis without physical separation.^15^ Crucially, our implementation achieves the depth required to simultaneously quantify phosphorylation dynamics, protein stability, and early newly synthesized proteomes from both compartments within a single experiment.

Our approach revealed coordinated yet distinct responses in melanoma cells and TILs during the early stages of T cell-mediated attack. In the immune compartment, we identified the cell surface receptor CRTAM as a marker of a tumor-reactive CTL subpopulation (Figure 5). Mechanistically, CRTAM interacts with its epithelial ligand Necl-2 (CADM1) on tumor cells to mediate strong cellular adhesion, a process that promotes NK cell cytotoxicity and enhances IFN-γ secretion by CD8^+^ T cells.^56^^,^^57^ Consistent with this, we showed that CRTAM expression is rapidly induced upon CTL stimulation and is associated with the secretion of IFN-γ. Clinically, CRTAM could be explored as a biomarker to stratify melanoma patients by the activity of tumor-reactive T cells, aiding prediction of ICB responses or monitoring the efficacy of adoptive T cell therapies. Moreover, therapeutically driving the upstream pathways that promote CRTAM-driven reactivity may offer a strategy to enhance overall CTL cytotoxicity in the TME.

In the tumor compartment, we observed rapid activation of kinases linked to the DNA damage response, including DNA-dependent protein kinase (DNA-PK/PRKDC: Figure 7). While DNA-PK is best known for its role in non-homologous end joining during DNA repair,^58^ it also acts as a STING-independent innate immune DNA sensor.^59^ Furthermore, inhibiting DNA-PK increases tumor MHC-I expression, an effect that synergizes with STING agonists to profoundly expand the neoantigen landscape and enhance TIL infiltration *in vivo*.^60^ Our detection of DNA-PK activation within 2 h of T cell engagement suggests that it is part of an immediate adaptive response to immune attack. Together with published data, these results support targeting DNA-PK as a strategy to impair tumor survival pathways and enhance tumor immunogenicity, potentially creating a therapeutic window for combination with immunotherapies.

Beyond protein phosphorylation and *de novo* synthesis, our method also analyzed targeted protein degradation (Figures 4C and 4D), which may serve as a rapid regulatory mechanism during early tumor-immune interactions. For instance, the degradation of JAK2 in melanoma cells upon co-culture may reflect an acute strategy to transiently suppress IFN-γ signaling, a hypothesis supported by clinical observations linking JAK2 loss-of-function mutations to acquired resistance to PD-1 blockade.^61^ Similarly, the degradation of Notch1 and the stabilization of TYR likely help tumor cells resist T cell-mediated killing, as constitutive Notch1 signaling sensitizes tumors to immunotherapy,^36^ while TYR actively inhibits T cell anti-tumor activity.^62^ However, not all degradation events favor evasion; the degradation of TRAF3, a negative regulator of the NF-κB pathway, may conversely support immune recognition by promoting PD-L1-independent MHC-I expression^63^ and lowering the TNF cytotoxicity threshold.^64^ On the TIL side, the degradation of CD6, a known inhibitory receptor, may act to enhance T cell activation.^65^ Concurrently, the degradation of CTSC, a key activator of granzymes in cytotoxic lymphocytes, likely dampens T cell activation,^66^ illustrating a highly dynamic, fine-tuning of the T cell response.

Ultimately, this framework provides a broadly applicable tool for dissecting dynamic interactions in intact mixed-cell systems where conventional isolation might disrupt labile PTMs or introduce technical bias. By mapping these high-resolution signaling events, our workflow opens new opportunities for biomarker discovery and therapeutic targeting. Integrating emerging multiplexing strategies will extend this methodology to more complex clinical models, bridging mechanistic discovery with translational cancer immunology.

### Limitations of the study

While our integrated approach of combining a 2D co-culture of patient-derived melanoma cells and autologous TILs with triple-SILAC quantitative proteomics provides a controllable platform to dissect direct tumor-T cell interactions, the overall methodology has multiple limitations. First, our *in vitro* model lacks most immune cell populations, three-dimensional architecture, extracellular matrix, and stromal components such as fibroblasts, all of which can shape immune responses. Several clinically relevant immune checkpoint pathways showed little or no functional effect in similar systems (Figure S7E), highlighting the need to validate candidate biomarkers and targets in more physiologically relevant models, including organoid-based co-cultures or mouse models. Second, in our metabolic labeling strategy, newly synthesized proteins during co-culture are incorporated into the medium-heavy SILAC channel without retaining information about the cell type of origin, meaning cell-type specificity is preserved only for pre-existing proteins. This introduces a time-dependent constraint: as co-culture progresses and more proteins are synthesized, a larger fraction of the proteome shifts into the medium-heavy channel, progressively reducing our ability to assign them to a specific cell type. Third, differences in cell size and baseline protein content result in lower proteomic depth for the smaller immune cells compared to the melanoma cells at the chosen E:T ratio of 1:1. Fourth, the triple SILAC labeling limits the approach to studying a maximum of two cell types, as all three isotopic states are required to distinguish compartments and track new protein synthesis. Fifth, despite the deep coverage we achieved, a significant fraction of the proteome and phosphoproteome remains analytically inaccessible, comprising the so-called dark proteome and phosphoproteome.^67^ This missing fraction is driven by inherent technical limitations, including a wide dynamic range masking low-abundance proteins,^68^ extraction biases against hydrophobic transmembrane or nuclear proteins,^69^ and non-optimal tryptic digestion.^70^ Furthermore, phospho-enrichment inherently favors certain phospho-motifs over others.^71^ Consequently, the absence of specific targets in our dataset reflects analytical non-detection rather than definitive biological absence. Sixth, our data analysis strategy was designed to evaluate each time point independently rather than as a continuous kinetic curve. In our co-culture system, the TILs have a lower baseline protein content per cell compared to the melanoma cells. To maintain high quantitative precision for this smaller immune cell population across independent replicates, we utilized sample-specific normalization and processed each time point in separate search batches. While this approach effectively minimizes technical variance within each time point, it does not support direct quantitative normalization or comparative analysis across different time points. Therefore, the current processed data should not be used for protein turnover analysis.

## Resource availability

### Lead contact

Requests for further information and resources should be directed to and will be fulfilled by the lead contact, Jesper V. Olsen (jesper.olsen@sund.ku.dk).

### Materials availability

This study did not generate new, unique reagents.

### Data and code availability

• Raw mass spectrometry data generated in this study have been deposited to the ProteomeXchangeConsortium^72^ via the MassIVE partner repository^73^ with the dataset identifiers PXD068403, PXD068582, and PXD068650. All processed data have been deposited to Zenodo at https://doi.org/10.5281/zenodo.18647598. All raw and processed data are publicly available as of the date of publication. For a complete list of all dataset accession codes and persistent identifiers (DOIs), also refer to the key resources table.
• To facilitate easy exploration of the data, an interactive web application has been developed. Users can query individual proteins to visualize both proteome and phosphoproteome expression levels across all patients and channels without downloading the datasets, accessible at https://giu-f.github.io/Melanoma_Proteomics/.
• All original code used for data analysis, as well as the source code for the web application, has been deposited on GitHub (https://github.com/Giu-F/Melanoma_Proteomics) and is permanently archived on Zenodo at https://doi.org/10.5281/zenodo.19691676.
• Any additional information required to reanalyze the data reported in this work paper is available from the lead contact upon request.

## Acknowledgments

This work was supported by the 10.13039/501100009708Novo Nordisk Foundation through the Exploratory Interdisciplinary Synergy Programme (grant NNF20OC0064594 to J.V.O. and M.D.) and core funding for the Center for Protein Research (grants NNF14CC0001 and NNF24SA0098829 to J.V.O.). Additional support was provided by the 10.13039/501100001732Danish National Research Foundation via a center-of-excellence grant to the Copenhagen Center for Glycocalyx Research (grant DNRF196 to J.V.O.) and the Danish Agency of Higher Education and Science for the PLATO research infrastructure (grant 5229-00012B to J.V.O.).

We are grateful to all patients who donated their samples for this work. We also thank Aishwarya Gokuldass for her participation in the early stages of this project, Anne-Christine Kiel Rasmussen for performing the melanoma flow cytometry panel experiments, Ulises Hernández Guzmán for helping with MS analysis of SILAC-labeled samples, Edoardo Dionisio for helping with the CRTAM follow-up experiments, Pierre Sabatier for helping designing the EGF experiment, and Per Thor Straten for donating the PBMCs.

## Author contributions

G.F. and J.V.O. designed the study. G.F. and A.W.P.J. optimized the SILAC DIA methodology, wrote the original draft of the manuscript, and generated the figures. G.F., A.W.P.J., and I.P. performed experiments. G.F., A.W.P.J., and A.M.-V. analyzed data. G.F. generated the R code used to analyze the data. J.V.O. and M.D. provided resources and coordinated the project. G.F., M.D., and J.V.O. supervised the project and acquired funds. All co-authors read and edited the manuscript.

## Declaration of interests

The authors declare no competing interests.

## Declaration of generative AI and AI-assisted technologies in the writing process

During the preparation of this work, the authors used ChatGPT and Gemini in order to improve language and readability. After using these tools, the authors reviewed and edited the content as needed and take full responsibility for the content of the publication.

## STAR★Methods

### Key resources table

**Table:** 

REAGENT or RESOURCE	SOURCE	IDENTIFIER
**Antibodies**		

Anti-CD3 (clone OKT3)	Miltenyi Biotec	Cat#130-093-377; RRID:AB_1036126
Anti-MCSP (PE, clone EP-1)	Miltenyi Biotec	Cat#130-129-293; RRID:AB_2922032
Anti-CD146 (BV421, clone P1H12)	BioLegend	Cat#361003; RRID:AB_2562966
Anti-CD90 (FITC, clone 5R10)	BioLegend	Cat#328107; RRID:AB_893438
Anti-CD107a (BV421, clone H4A3)	BD Biosciences	Cat#562623; RRID:AB_2737685
Anti-CD3 (BV786, clone SK7)	BD Biosciences	Cat#563800; N/A
Anti-CD8 (Qdot™ 605, clone 3B5)	Invitrogen, Thermo Fisher Scientific	Cat#Q10009; RRID:AB_2556437
Anti-CD8 (APC-R700, clone RPA-T8)	BD Biosciences	Cat#565165; RRID:AB_2744457
Anti-CD4 (BV510, clone SK3)	BD Biosciences	Cat#562970; RRID:AB_2744424
Anti-CRTAM (PE, clone Cr24.1)	BioLegend	Cat#339106; RRID:AB_2085907
Anti-CD137 (BV605, clone 4B4-1)	BioLegend	Cat#309822; RRID:AB_2565997
Anti-CD137 (APC, clone 4B4-1)	BioLegend	Cat#309810; RRID:AB_830672
Anti-TNFα (APC, clone MAb11)	BioLegend	Cat#502912; RRID:AB_315264
Anti-IFNγ (PE-Cy7, clone B27)	BD Biosciences	Cat#557643; RRID:AB_396760
Anti-CD25 (PE-Cy7, clone 2A3)	BD Biosciences	Cat#335824; RRID:AB_2868687

**Biological samples**		

Healthy donor blood samples	Odense Hospital, Denmark	N/A
Human metastatic melanoma tumor biopsies	CCIT-DK, Herlev Hospital, Denmark	N/A

**Chemicals, peptides, and recombinant proteins**		

Dulbecco’s phosphate-buffered saline (PBS)	Gibco, Thermo Fisher Scientific	Cat#10-010-023
RPMI-1640 medium	Gibco, Thermo Fisher Scientific	Cat#72400047
RPMI-1640 medium for SILAC	Thermo Fisher Scientific	Cat#88365
DMEM/F12 medium	Gibco, Thermo Fisher Scientific	Cat#31331093
AIM-V medium	Gibco, Thermo Fisher Scientific	Cat#12055083
Fetal bovine serum (FBS)	Gibco, Thermo Fisher Scientific	Cat#10270106
Dialyzed fetal bovine serum (dFBS)	Gibco, Thermo Fisher Scientific	Cat#26400044
Human serum	Sigma-Aldrich, Merck	Cat#H4522
Penicillin-Streptomycin	Gibco, Thermo Fisher Scientific	Cat#15140122
IL-2 (Proleukin) 22 × 10e6 IU/vial	Novartis provided by Danish hospital pharmacy	N/A
Recombinant IFN-γ	Peprotech	Cat#300–02
Epidermal growth factor (EGF)	Peprotech	Cat#AF-100-15
Dynabeads Human T-Activator CD3/CD28	Gibco, Thermo Fisher Scientific	Cat#11132D
PMA/Ionomycin	Invitrogen, Thermo Fisher Scientific	Cat#00-4970-93
Sodium orthovanadate	Sigma-Aldrich, Merck	Cat#S6508
Sodium fluoride	Sigma-Aldrich, Merck	Cat#S7920
β-glycerophosphate	Sigma-Aldrich, Merck	Cat#G9422
Sodium dodecyl sulfate (SDS)	Sigma-Aldrich, Merck	Cat#05030
Tris(2-carboxy- ethyl)phosphine (TCEP)	Sigma-Aldrich, Merck	Cat#C4706
Chloroacetamide (CAA)	Sigma-Aldrich, Merck	Cat#C0267
Hydrochloric acid, 37%	Sigma-Aldrich, Merck	Cat#320331
Trizma base	Sigma-Aldrich, Merck	Cat#T1503
Trifluoroacetic acid (TFA)	Sigma-Aldrich, Merck	Cat#8082600501
Acetonitrile (ACN)	Sigma-Aldrich, Merck	Cat#1000302500
Formic acid	Thermo Fisher Scientific	Cat#28905
Lys-C	FUJIFILM Wako Pure Chemical Corporation	Cat#129-02541
Trypsin	Sigma-Aldrich	Cat#T6567
Zr-IMAC HP beads	ReSyn Biosciences	Cat#MR-ZHP
Ti-IMAC HP beads	ReSyn Biosciences	Cat#MR-THP
L-Lysine-2HCl (13C6 15N2)	Cambridge Isotope Laboratories	Cat#CNLM-291-H
L-Arginine-HCl (13C6 15N4)	Cambridge Isotope Laboratories	Cat#CNLM-539-H
L-Lysine-2HCl (D4)	Cambridge Isotope Laboratories	Cat#DLM-2640-O
L-Arginine-HCl (13C6)	Cambridge Isotope Laboratories	Cat#CLM-2265-H
Live/Dead™ Fixable Near-IR (APC-Cy7)	Thermo Fisher Scientific	Cat#L34976
Brefeldin A (GolgiPlug)	BD Biosciences	Cat#555029
Monensin (GolgiStop)	BD Biosciences	Cat#54724

**Critical commercial assays**		

Permeabilization Buffer	eBiosciences, Thermo Fisher Scientific	Cat#00-8333-56
Fixation/Permeabilization Concentrate	eBiosciences, Thermo Fisher Scientific	Cat#00-5123-43
Fixation/Permeabilization Diluent	eBiosciences, Thermo Fisher Scientific	Cat#00-5223-56
BCA Protein Assay Kits	Pierce™, Thermo Fisher Scientific	Cat#23225

**Deposited data**		

Raw data	This paper	ProteomeXchangeConsortium^72^ via the MassIVE partner repository^73^ with the dataset identifiers PXD068403, PXD068582 and PXD068650.
Analyzed data	This paper	https://doi.org/10.5281/zenodo.18647598
R code	This paper	https://github.com/Giu-F/Melanoma_Proteomics & https://doi.org/10.5281/zenodo.19691676
CRISPR-KO data	N/A	Kearney et al.^31^ and Zhang et al.^32^
Single-cell RNA sequencing data	Broad Institute; https://singlecell.broadinstitute.org/single_cell/study/SCP109/melanoma-immunotherapy-resistance	RRID:SCR_014816; Jerby-Arnon et al.^38^
UniProt human proteome database	UniProt	Downloaded October 2024

**Experimental models: Cell lines**		

SCC-25	ATCC	Cat#CRL-1628

**Software and algorithms**		

DIA-NN v2.0	https://github.com/vdemichev/DiaNN	RRID:SCR_022865
R v4.4.1	The R Project for Statistical Computing; https://www.r-project.org/	RRID:SCR_001905
RStudio v2024.12.1	Posit PBC; https://posit.co/download/rstudio-desktop/	RRID:SCR_000432
Limma (R package)	Bioconductor	Ritchie et al.^74^
KEGGREST (R package)	Bioconductor	RRID:SCR_026949
Arrow (R package)	CRAN	N/A
Protti (R package)	CRAN	N/A
Data.table (R package)	CRAN	RRID:SCR_026117
Tidyverse (R package)	CRAN	RRID:SCR_014601
Pheatmap (R package)	CRAN	RRID:SCR_016418
Eulerr (R package)	CRAN	RRID:SCR_022753
Cytoscape	https://cytoscape.org	RRID:SCR_003032
STRING	STRING app	RRID:SCR_005223; Doncheva et al.^75^
Omics Visualizer	Omics Visualizer app	RRID:SCR_018077; Legeay et al.^76^
PTMNavigator	https://www.proteomicsdb.org/analytics/ptmNavigator	Müller et al.^55^
RoKAi v2.3.0	https://rokai.io/	Yilmaz et al.^51^
NovoExpress v1.5.6	Agilent	RRID:SCR_024676
RTCA eSight Software Basic v1.1.1	Agilent	RRID:SCR_019571
Chromeleon	Thermo Fisher Scientific	RRID:SCR_016874
Kaplan-Meier Plotter	https://kmplot.com/analysis/	RRID:SCR_018753
TIDE	Before: http://tide.dfci.harvard.edu. Now: https://cide.ccr.cancer.gov/	N/A
pLogo	https://plogo.uconn.edu/	RRID:SCR_018185; O'Shea et al.^48^
GraphPad Prism v10	https://www.graphpad.com/	RRID:SCR_002798

**Other**		

C18 Sep-Pak cartridges	Waters Corporation	Cat#186002320
EvoTip Pure	Evosep Biosystems	EV2011

### Experimental model and study participant details

#### Sample origin

Tumor biopsies were obtained from four patients diagnosed with cutaneous metastatic melanoma, enrolled in the clinical trials at the National Center for Cancer Immune Therapy (CCIT-DK), Department of Oncology, Copenhagen University Hospital, Herlev, Denmark (H-20070020). Peripheral Blood Mononuclear Cells (PBMCs) were obtained from healthy donors at CCIT-DK. All procedures were approved by the Scientific Ethics Committee for the Capital Region of Denmark. Written informed consent was obtained from patients before any procedure according to the Declaration of Helsinki.

The four samples included in the study were derived from two anti-PD1 therapy-naïve and two patients with confirmed resistance to anti-PD1 therapy (Table S2). Melanoma was chosen as a tumor model with known high TIL reactivity.^77^

#### Establishment of primary melanoma cell lines and REP TILs

From each biopsy, a matched pair of primary melanoma cells and autologous TILs were established *in vitro*, as described elsewhere.^78^^,^^79^ Briefly, tumor specimens were obtained fresh and immediately transported to the laboratory in RPMI 1640 (Gibco, Thermo Fisher Scientific). The tumor masses were isolated from the surrounding tissues and sliced into multiple fragments (1–3 mm^3^ each) with a scalpel. Patient-derived melanoma cell lines were established from tumor fragments through short-term *in vitro* serial passages of adherent cells. After establishment, they were authenticated based on growth pattern, morphology and flow cytometry characterization analysis (described below). TILs were established *in vitro* by a two-step expansion process. First, they were “minimally expanded”^80^ in high doses of IL2 (6,000 IU/mL) (Proleukin, Novartis) from tumor fragments. When a minimum of 50 × 10^6^ TILs were obtained (typically about 14–28 days after surgical resection), expansion was further achieved by a 14-day “rapid expansion” protocol (REP), in which TILs were unspecifically expanded with a 200-fold excess of allogeneic irradiated peripheral blood mononuclear cells (PBMCs) from healthy donors, high doses of IL2 and 30 ng/mL of anti-CD3 antibodies (clone OKT3, Miltenyi Biotec). The composition and functional reactivity of the REP TIL batches were subsequently analyzed via flow cytometry (described below).

#### Cell culture, ligand stimulation and drug treatment

All cells were cultured at 37°C in a humidified incubator with 5% CO2 and tested monthly for mycoplasma contamination by PCR. Squamous Cell Carcinoma (SCC)-25 cells (male) were cultured in DMEM/H12 (Gibco, Thermo Fisher Scientific), supplemented with 10% fetal bovine serum (FBS, Gibco, Thermo Fisher Scientific), 100 U/ml penicillin and 100 μg/mL streptomycin (Pen-Strep, Gibco, Thermo Fisher Scientific). Primary melanoma cells were cultured in RPMI 1640 Medium with GlutaMAX (Gibco, Thermo Fisher Scientific), supplemented with 25 mM HEPES (Gibco, Thermo Fisher Scientific), 10% FBS and Pen-Strep. REP TILs were cultured in RPMI-1640 with GlutaMAX, supplemented with 25mM HEPES, 10% heat-inactivated human AB serum (HS, Sigma-Aldrich/Merck) and Pen-Strep; or else in AIM-V (Gibco, Thermo Fisher Scientific), supplemented with 10% HS and 6000 IU/mL of IL2. PBMCs were cultured in X-VIVO (Lonza) supplemented with 5% HS and 100 IU/mL of IL2.

SCC-25 were stimulated with 100 ng/mL of epidermal growth factor (EGF; Preprotech) for 8 min. Next, the cells were washed with PBS, trypsinized and centrifuged to obtain a cell pellet that was either lysed right away ("Immediately-Lysed", IL) or incubated in PBS on ice for 3 h with or without the following phosphatase inhibitors (PI): 5 mM sodium orthovanadate, 1 mM sodium fluoride and 1 mM beta-glycerophosphate. REP TILs were stimulated with: CD3/CD28 beads (Dynabeads Human T-Activator CD3/CD28, Gibco, Thermo Fisher Scientific) for 30 min, 6 or 8 h at 1 bead per 2 TILs ratio; CD3 (OKT3) antibody for 6 h at a concentration of 30 ng/mL; 100 international units (IU) of recombinant interferon-γ (IFN-γ) for 6 h; PMA/Ionomycin for 30 min, 6 or 8 h at a concentration of PMA 25 ng/mL and Ionomycin 0.5 μM. Melanoma cells were stimulated with 100 IU of IFN-γ for 6 or 24 h. PBMCs were stimulated with CD3/CD28 beads at 1:2 ratio.

#### SILAC labeling of melanoma cells

Patient-derived melanoma cell lines were cultured in SILAC RPMI 1640 with GlutaMAX (Thermo Fisher Scientific), 10% dialyzed fetal bovine serum (dFBS, Gibco, Thermo Fisher Scientific), Pen-Strep and 0.028 mg/mL of heavy L-Lysine-2HCl (^13^C_6_^15^N_2_, Cambridge isotope laboratories) and 0.049 mg/mL of heavy L-Arginine-HCl (^13^C_6_^15^N_4_, Cambridge isotope laboratories). After 10–14 days, heavy isotope incorporation was assessed using MS. The resulting cell batches were cryopreserved for future use in co-culture experiments for proteomics analysis.

### Method details

#### Flow cytometry analysis

Cells were analyzed on a NovoCyte Quanteon Flow Cytometer. Wells from the same plate were considered technical replicates, while measurements performed on different days were considered biological replicates. All experiments were performed in three technical replicates.

##### Melanoma cells

For melanoma cells, 5 × 10^5^ cells were collected and washed twice with Dulbecco’s phosphate-buffered saline (PBS, Gibco, Thermo Fisher Scientific). Cells were then stained with Live/Dead Fixable Near-IR (APC-Cy7, Thermo Fisher Scientific) and subsequently for anti-MCSP (PE, clone EP-1, Miltenyi Biotec), anti-CD146 (BV421, clone P1H12, BioLegend) and anti-CD90 (FITC, clone 5R10, BioLegend) antibodies in PBS with 0.1% FBS at 4°C for 30 min, protected from light. After staining, cells were washed twice in PBS.

##### Activated TILs

Tumor-specific immune reactivity of REP TILs was assessed with 6 or 8-h co-culture assays at 37°C with an effector:target (E:T) ratio of 1:1 or 3:1. When assessing degranulation and cytokine production, the antibody anti-CD107a (BV421, Clone H4A3, BD Biosciences) was added prior to co-culture with brefeldin A (1:1,000, GolgiPlug, BD Biosciences) and monensin (1:1,000, GolgiStop, BD Biosciences). In experiments involving simultaneous detection of both intracellular cytokines and CRTAM, only monensin was added. REP TILs cultured alone or with PMA/Ionomycin served as negative and positive control, respectively. After co-culture, REP TILs were stained with Live/Dead Fixable Near-IR (APC-Cy7, Thermo Fisher Scientific) and the following antibodies for 30 min: anti-CD3 (BV786, clone SK7, BD Biosciences), anti-CD8 (Qdot 605, clone 3B5, Invitrogen; APC-R700 clone RPA-T8, BD Horizon), anti-CD4 (BV510, clone SK3, BD Biosciences), anti-CRTAM (PE, clone Cr24.1 BioLegend), anti-CD137 (APC, clone 4B4-1; BioLegend), and CD25 (PE-Cy7, clone 2A3, BD Biosciences). The cells were then washed twice with PBS, fixed and permeabilized overnight at 4°C using the FoxP3/Transcription Factor Staining Buffer Set (eBiosciences, Thermo Fisher Scientific). The following day, the cells were stained with anti-CD137 (BV605, clone 4B4-1, BioLegend), anti-TNFα (APC, Clone MAb11, Invitrogen) and anti-IFNγ (PE-Cy7, Clone B27, BD Biosciences).

#### Proliferation assay on PBMCs

PBMCs from a healthy donor were labeled with CellTrace Violet (Thermo Fisher Scientific) and stimulated with CD3/CD28 beads. Cells were subsequently stained with Live/Dead Fixable Near-IR and antibodies against CD3 (BV786), CD8 (Qdot 605), and CRTAM (PE) prior to flow cytometric acquisition.

#### Real-time tumor killing analysis using xCELLigence

The cytotoxicity of REP TILs against tumor cells was assessed using a real-time cell analysis (RTCA) assay on the xCELLigence RTCA eSight system (Agilent), according to the manufacturer’s instructions. Briefly, tumor cells were seeded into a 96-well RTCA E-plate and incubated for 24 h, until reaching a Cell Index (CI) value of 1. The day prior to the co-culture experiment, REP TILs were thawed and rested overnight in TILs media. On the day of the experiment, half of the tumor medium was replaced with either fresh medium (control) or medium containing autologous REP TILs. CI measurements were recorded hourly for 72 h after the addition of REP TILs, using the RTCA eSight Software Basic version 1.1.1 (Agilent).

Wells from the same plate were considered technical replicates, while measurements performed on different days were considered biological replicates. For all experiments, each experimental condition was performed in at least 3 technical replicates.

#### Co-culture of heavy-labeled melanoma cells with autologous REP TILs for proteomic and phosphoproteomic analysis

Prior to the co-culture experiment, REP TILs were thawed and rested overnight in TILs media supplemented with 6000 IU/mL of IL2. The following day, REP TILs were short-term-expanded in AIM-V supplemented with 10% HS and 6000 IU/mL of IL2 for 5 to 7 days. Two days before the co-culture, 1.5 × 10^6^ SILAC-heavy labeled tumor cells were seeded in a 10 cm dish in SILAC-heavy medium. After 24 h, SILAC-heavy medium was replaced with serum-reduced SILAC-heavy medium (1% dFBS), while REP TILs were starved in AIM-V, supplemented with 50 IU/mL of IL2. After 15 h of starvation, 2 × 10^6^ of seeded tumor cells per replicate were washed with 37°C PBS, followed by incubation with serum-reduced SILAC medium-heavy medium [SILAC RPMI 1640 with GlutaMAX, 10% dFBS, Pen-Strep, 0.028mg/mL of L-Lysine-2HCl (4,4,5,5-D4, Cambridge isotope laboratories) and 0.049mg/mL L-Arginine-HCl (^13^C_6_, Cambridge isotope laboratories) with 2 × 10^6^ REP TILs (ET ratio 1:1). As a control, tumor cells and TILs were incubated separately, and then reunited after lysis. Samples were harvested before co-culture (0 h), and after 2 and 6 h. 2 and 6 × 10^6^ of tumor cells and REP TILs per replicate, respectively, were processed for proteomic analysis at time 0 h as monoculture. REP TILs were recovered from the supernatant, washed in PBS and centrifuged to obtain a cell pellet. Tumor cells were washed with PBS and lysed while still adhering on the plate.

For the proteomics experiment, each condition per patient was performed in six biological replicates (one replicate = one dish). Replicates were generated in close succession, within approximately one week. All samples downstream of cell lysis were processed and acquired together.

#### Generation of a project-specific phosphopeptide spectral library

To generate a project-specific phosphopeptide spectral library, for each individual patient the co-culture experiment was also performed in one biological replicate with unlabeled (light) melanoma cells. Time points 2 and 6 h were pulled. These samples were further processed for phosphoproteomic analysis (see below), and then processed with two different workflows.

1. High-pH reversed-phase peptide fractionation, followed by phospho-enrichment and MS analysis (only for patient 905).
2. Phospho-enrichment, followed by MS analysis by gas-phase fractionation (for all patients).

Only for patient 905, we generated two additional sets of samples, using TILs in monoculture stimulated with CD3/CD28 beads or PMA/Ionomycin for 30 min. These samples were only processed with workflow 1.

#### Sample preparation for proteomic and phosphoproteomic analysis

##### Cell lysis and protein extraction

Cells were lysed with boiling lysis buffer [5% sodium dodecyl sulfate (SDS), 5 mM tris(2-carboxyethyl)phosphine (TCEP), 10 mM chloroacetamide (CAA), 100 mM Tris HCl pH 8.5] and incubated for 10 min at 99°C while shaking. Samples were sonicated and protein concentration was determined using a BCA assay (Pierce).

##### Protein digestion

For each sample, 50–300 μg of protein was digested overnight with Lys C (FUJIFILM Wako Pure Chemical Corporation) and Trypsin (Sigma-Aldrich) at an enzyme:protein ratio of 1:500 and 1:250, respectively, using the KingFisher magnetic particle separation robot using the optimized protocol explained in detailed elsewhere.^81^^,^^82^ The following day, samples were acidified with trifluoroacetic (TFA) to a final concentration of 1%. 0.5–1 μg of peptides were loaded on Evotips (Evosep Biosystems) for proteome analysis. The rest of the peptides were further processed by desalting with solid-phase extraction using C18 Sep-Paks (Waters Corporation), followed by speedvac until acetonitrile (ACN) evaporation. The final peptide concentration was estimated by measuring absorbance at 280 nm on a NanoDrop 2000C (Thermo Scientific), before proceeding with Ti-IMAC phosphopeptide enrichment.

##### High-pH reversed-phase peptide fractionation

To generate a project-specific phospho-enriched spectral library for patient 905, 200 μg of unlabeled peptides were resuspended in 20 μL of 25 mM ABC and fractionated using a reversed-phase Acquity CSH C18 1.7 μm × 1 mm × 150 mm column (Waters) coupled to the UltiMate 3000 high-performance liquid chromatography (HPLC) system (Thermo Fisher Scientific) by using the Chromeleon software (Thermo Fisher Scientific). The instrument operated at 30 μL/min with column oven temperature set to 40 °C. Buffer A (5 mM ABC) and buffer B (100% ACN) were used. Peptides were eluted in 12 fractions with concatenation via multi-step gradient as follows: 0–62.5 min 8-28% B; 62.5–67 min 28-60% B; 67–70 min 60-70% B; 70–77 min 70% B; 77–78 min 8% B; 78–87 min 8% B. Fractions were acidified with formic acid (FA; 40 μL of 10% FA) to a final concentration of 1% and phosphorylated peptides were enriched by Ti-IMAC enrichment, with a peptide input per fraction of 17 μg.

##### Enrichment of phosphorylated peptides

Ti-IMAC phosphopeptide enrichment was carried out on a KingFisher Flex robot (Thermo Fisher Scientific) in 96-well format, as previously described.^26^^,^^81^^,^^82^ Peptide input amounts were dictated by the specific experimental purpose. For all quantitative co-culture experiments and HPF libraries, the peptide input was 17 μg per sample. For the Phospho-Alanine decoy experiment, the peptide input was 20 μg per sample. For the generation of GPF libraries, higher peptide amounts were utilized per fraction, varying based on total sample availability (40, 60.5, 102.8 and 131.7 μg for patients 905, 915, 924 and 605, respectively). For the enrichment, magnetic Zr-IMAC HP beads (ReSyn Biosciences) were used at a ratio of 10 μL of bead slurry per 100 μg of peptide, with a minimum bead volume of 5 μL per reaction. Eluted phosphopeptides were acidified with 40 μL of 10% TFA and loaded on Evotips (Evosep Biosystems) for MS analysis.

#### LC-MS/MS analysis

LC-MS/MS analysis was performed on an Orbitrap Astral mass spectrometer (Thermo Fisher Scientific)^16^ coupled to a Vanquish *Neo* UHPLC (Thermo Fisher Scientific) in the trap-and-elute mode or an Evosep One LC system (Evosep Biosystems),^83^ and interfaced online using an EASY-Spray source. The analytical column type was chosen according to the experiment (Table S7).

The Orbitrap Astral mass spectrometer was operated in positive ion mode with data-independent acquisition (DIA). The spray voltage was set at static, 1.8 or 2 kV, the heated capillary temperature at 275 or 280°C and funnel RF frequency at 40. The full-MS resolution was set at 120K, 180K or 240K with a full scan range of m/z 380–980 for proteome and m/z 480–1080 for phosphoproteome. For the gas-phase fractionation library, each sample was split in 3 fractions, and the precursor scan range was 480–680, 680–880 and 880–1080, respectively. The full-MS automatic gain control (AGC) was set to 500% and the maximum injection time was set at 3, 5, 10 or 30 ms. The isolated peptide precursor ions were fragmented using HCD with 25% Normalized Collision Energy (NCE). The fragment scan range was set at 150–2000 m/z. DIA-MS/MS fragment ion scans were recorded with a 1, 2, 4 or 6 Th quadrupole isolation window and a maximum injection time of 2.5, 3, 6, 8 or 12 ms. The MS method details for each experiment are described in Table S7.

### Quantification and statistical analysis

#### Flow cytometry data analysis

Data was analyzed with NovoExpress software version 1.5.6 (Agilent). Gates were defined using unstimulated TILs and/or fluorescence minus one (FMO) controls. Initial gating was performed using forward scatter area (FSC-A) and side scatter area (SSC-A) to exclude cellular debris and isolate the general intact cell population. Doublet discrimination was then performed by plotting FSC-A against forward scatter height (FSC-H) to gate on single cells. Dead cells were excluded using a near-infrared viability dye (NIR-APC-Cy7-A), allowing for the isolation of the live cell population. From the live single-cell gate, total T cells were identified by gating on CD3-positive events (CD3-BV786-A). This CD3^+^ population was further subdivided into CD8^+^ and CD4^+^ T cell subsets using CD8-APC-R700-A and CD4-BV510-A, respectively.

#### XCELLigence data analysis

CI was normalized to the time point where REP TILs were added, to obtain the Normalized Cell Index (NCI). The percentage of tumor cell killing was calculated by dividing the NCI of the co-culture by the tumor alone and multiplied by 100.

#### Raw mass spectrometry data processing

Raw MS data were analyzed using DIA-NN,^24^ version 2.0. The Human Uniprot fasta file was downloaded in October 2024 and contained 20,428 entries.

Three spectral libraries (one for proteome, one for phosphoproteome and one for the Phospho-Alanine decoy search) were generated *in-silico* by enabling “FASTA digest for library-free search/library generation” and “Deep learning-based spectra, RTs and IMs prediction”. No raw data was supplied in this step. For the proteome library, the settings were the following: maximum number of missed cleavages set to 1, maximum number of variable modifications set to 0, N-terminal methionine excision enabled, cysteine carbamidomethylation enabled as a fixed modification, minimum peptide length set to 7, maximum peptide length set to 30, minimum precursor charge set to 2, maximum precursor charge set to 4, minimum precursor m/z set to 380, maximum precursor m/z set to 980, minimum fragment m/z set to 150, max fragment m/z set to 2000, contaminants enabled. For the phosphoproteome library, the settings were the following: maximum number of missed cleavages set to 2, maximum number of variable modifications set to 3, N-terminal methionine excision enabled, cysteine carbamidomethylation enabled as a fixed modification, phosphorylation on STY enabled as variable modification, minimum peptide length set to 7, maximum peptide length set to 30, minimum precursor charge set to 2, maximum precursor charge set to 4, minimum precursor m/z set to 480, maximum precursor m/z set to 1080, minimum fragment m/z set to 150, max fragment m/z set to 2000, contaminants enabled. For the Phospho-Alanine decoy library, the settings were the following: maximum number of missed cleavages set to 1, maximum number of variable modifications set to 2, N-terminal methionine excision enabled, cysteine carbamidomethylation enabled as a fixed modification, phosphorylation on STYA enabled as variable modification (--var-mod UniMod:21,79.966331,STY typed in “Additional options”), minimum peptide length set to 7, maximum peptide length set to 30, minimum precursor charge set to 2, maximum precursor charge set to 4, minimum precursor m/z set to 480, maximum precursor m/z set to 1080, minimum fragment m/z set to 150, max fragment m/z set to 2000, contaminants enabled.

The “in-silico” spectral libraries were supplied to search raw MS data to generate empirical spectral libraries with the same settings used for the predicted libraries, except for “FASTA digest for library-free search/library generation” and “Deep learning-based spectra, RTs and IMs prediction” that were unchecked. The command “generate spectral library” was enabled and “MBR” was disabled. Mass accuracy and scan window were fixed (values depend on the experiment: see Table S7). For the search of the project-specific phospho-enriched fractionated spectral library, “Unrelated runs” was also enabled.

When searching SILAC raw files, the following text was added to “Additional options”:

--fixed-mod SILAC,0.0,KR,label.

--lib-fixed-mod SILAC.

--channels SILAC,L,KR,0:0; SILAC,M,KR,4.025107:6.020129;SILAC,H,KR,8.014199:10.008269.

--original-mods.

--channel-spec-norm.

Since the --channel-spec-norm command normalizes data within a given time point, the resulting search output does not support direct quantitative comparisons across different time points.

The obtained empirical spectral libraries were supplied to search raw MS data separately for each time point and MS method, using the same settings discussed above. Importantly, since the proteome empirical spectral library was derived from multiplexed runs, “--lib-fixed-mod SILAC” was not added to “Additional options”.

For the SCC-25 phosphoproteome and the IFN-γ proteome, the search was performed against the corresponding empirical libraries with MBR enabled.

#### Bioinformatic analysis

All bioinformatic analysis was performed using R version 4.4.1 with R studio version 2024.12.1.

##### Proteomics data filtering and processing

For all SILAC datasets, the main report in ".parquet" format was used and processed with the R package “arrow”. For the IFN-γ proteome dataset, “pg_matrix.tsv” was used. For the SCC-25 phosphoproteome dataset, “phosphosites_90.tsv” was used.

For all datasets, contaminants and entries without a gene name were removed, and MS intensities were log2 transformed.

For all SILAC datasets, entries without a channel were removed. Data were filtered at 1% FDR, using global q-values for protein groups (“Lib.PG.Q.Value” ≤ 0.01) and both global and run-specific q-values for precursors (“Q.Value” and “Lib.Q.Value” ≤ 0.01, respectively). An additional 5% run-specific protein-level FDR filter (“PG.Q.Value” ≤ 0.05) was applied too.

For SILAC proteome datasets, only protein entries with a “PG.MaxLFQ.Quality” ≥ 0.7 were kept. The column “PG.MaxLFQ” was used for downstream quantitative analyses.

For the SILAC phosphoproteome dataset, data were additionally filtered at 1% FDR using global peptidoform q-values (“Lib.Peptidoform.Q.Value” ≤ 0.01, respectively), and channel-level q-value (“Channel.Q.Value” ≤ 0.01). Only precursors with a “Quantity.Quality” ≥ 0.5 that were phosphorylated on a serine, threonine or tyrosine were kept. The column “Precursor.Normalised” was used for downstream quantitative analyses. Precursor-level quantifications were aggregated to the phosphosite level based on the “Protein.Sites” column, considering only phosphorylation events and excluding other modifications. Phosphosites detected on precursors with different phosphorylation multiplicities (i.e., singly, doubly, or triply phosphorylated) were not merged and were labeled accordingly: M1 for singly, M2 for doubly and M3 for triply phosphorylated precursors. Run-specific site localization probabilities were extracted from the “Site.Occupancy.Probabilities” column. Prior to aggregation, any phosphosite with a localization probability below 0.75 in a given run was excluded from the analysis. The aggregation was performed using the “top-1 method”: for each site, the precursor entry with the highest summed raw intensity across runs was retained. The sequence window was extracted from the protein sequence, including 5 amino acids before and after the phosphorylated residue. The protein sequence was retrieved with the R package “Protti”, using the function “fetch_uniprot”.

Labeling efficiency for the heavy channel was calculated at the time point 0 h for the tumor-alone samples as the number of protein groups identified in the heavy channel divided by the total number of protein groups identified across all channels.

Heavy channel false discovery rate (FDR) was calculated at the time point 0 h for the TILs-alone samples as the number of protein groups identified in the heavy channel divided by the total number of protein groups identified across all channels. Medium-heavy channel FDR was calculated at the time point 0 h separately for tumor- and TILs-alone samples as the number of protein groups identified in the medium-heavy channel divided by the total number of protein groups identified across all channels (Figure 2D).

To validate site localization accuracy, we used a decoy-residue entrapment strategy by searching for a “ghost” modification^11^ (Figures S2D and S2E), namely phospho-Alanine (pA). Since Alanine phosphorylation is not biologically expected, any high-confidence phospho-assignment to an Alanine represents a stochastic mislocalization. We calculated a normalized FLR to account for the fact that Alanine and STY frequencies differ in the proteome.^84^ The formula used was: FLR = [(Ndecoy+Ntarget)/Ndecoy] x [#D/(#T + #D)], where N is the total number of phosphorylated precursors in the predicted library for decoys (A) or target (STY); # is the actual number of identified decoy (D) or T (T) hits.

Protein group coefficient of variation (CV) was calculated only for the co-culture samples by dividing the standard deviation (SD) by the mean of raw MS intensity for entries with at least 3 valid values per channel.

Pearson correlation was calculated using pairwise complete observations.

Proteins belonging to the EGFR signaling pathway were retrieved using the R package “KEGGREST” as part of the KEGG pathway “ErbB signaling pathway” (hsa04012).

##### Presence/absence

To identify proteins and phosphosites that were exclusively present in one condition, we established identification thresholds. In the SILAC proteome dataset, a protein was considered uniquely present in the co-culture if it was detected in no more than 2 of the 48 control replicates and in at least 7 of the 48 co-culture replicates. Analogous criteria were applied to the IFN-γ proteome dataset (a maximum of 2/36 detections in controls and a minimum of 7/36 in treated samples) and the phosphoproteome dataset (a maximum of 2/24 detections in controls and a minimum of 10/24 in co-culture samples).

##### Differential expression analysis (DEA)

Data was further filtered before DEA.

• SCC-25 phosphoproteome: entries with at least 3 valid values in at least one experimental group (lL_Ctrl, IL_EGF, PBS_Ctrl, PBS_EGF, PI_Ctrl, PI_EGF) were included.
• IFN-γ proteome: proteins were retained if they contained at least 2 valid values in all 3 patients and 3 valid values for both the control and IFN-γ conditions at either the 6-h or 24-h time point.
• SILAC proteome and phosphoproteome: For the global analysis of both SILAC proteome and phosphoproteome datasets, filtering was performed separately on each channel. We included only entries that had 2 or more valid values in at least 3 patients, with a minimum of 3 valid values per experimental group (control and co-culture). Additionally, for the patient-specific analysis of the SILAC phosphoproteome, filtering was performed individually for each patient/channel; here, entries were required to have at least 2 valid values per experimental group within the respective patient.

DEA was performed using the limma package.^74^^,^^85^ We accounted for repeated measures by using the “duplicateCorrelation” function to estimate the correlation between samples from the same patient. This correlation was incorporated into the model via the “block” argument in the “lmFit” function.^86^ For the SCC-25 phosphoproteome analysis and the patient-specific analysis of the SILAC phosphoproteome, no blocks were set. Empirical Bayes moderation was applied using “eBayes” with “trend = TRUE”, which allows the prior variance estimate to depend on the average intensity of each protein or phosphosite.^85^ *p* values were corrected using the Benjamini Hochberg (BH) procedure. For all SILAC datasets, BH correction was performed separately for each channel. An entry was considered regulated if the adjusted *p* value was equal or smaller than 0.01.

A fold-change cutoff was also applied to define significant regulation across all datasets, adapted to the specific distribution of each experiment.

• SCC-25 phosphoproteome: the standard deviation (SD) was calculated separately for each contrast (IL, PBS, PI), and for up- and down-regulated phosphosites. This value multiplied by 2 ranged from 0.9 to 1. Therefore, a phosphosite was considered regulated if the log2 fold-change was equal or greater than 1 for up-regulation and −1 for down-regulation.
• IFN-γ proteome: the SD was calculated separately for each time point (6 and 24 h), and for up- and down-regulated proteins. This value multiplied by 2 was used as a fold-change cutoff (6 h: up-regulation = 0.24; down-regulation = −0.26; 24 h: up-regulation = 0.75; down-regulation = −0.37).
• SILAC datasets: Due to the distribution tailing toward the co-culture side, we employed the Median Absolute Deviation (MAD) instead of SD. MAD was calculated scaled to standard deviation separately for each channel, and for up- and down-regulated proteins/phosphosites.The final cutoff was determined as the average of the Up and Down variability thresholds. Proteome: Cutoff defined as 5x MAD. Phosphoproteome: Cutoff defined as 3x MAD. This resulted in cutoffs of 0.35 and 0.65 log2FC, respectively.

Proteins with differential degradation were defined as showing significant regulation in the heavy or light channels (representing the pre-existing proteome) that were not regulated in the same direction in the medium-heavy channel. The medium-heavy channel served as a control to monitor potential re-incorporation of unlabeled amino acids (recycling) into newly synthesized proteins. To strictly exclude proteins where abundance changes were driven by synthesis/recycling rather than pure degradation, we applied a loose exclusion filter: any protein displaying nominal statistical significance (*p* ≤ 0.05) in the medium-heavy channel, regardless of fold change magnitude, was flagged as a potential recycling artifact and removed from the differential degradation candidate list.

##### Differential regulation of heavy vs. light channels and functional annotation of phosphosites

To identify phosphosites that were differentially regulated between the melanoma and TILs (Channel H vs. Channel L; Figure 6), we compared the log2 fold-changes (logFC) obtained from the limma analysis. Phosphosites identified as significantly regulated in at least one channel were extracted, and their logFC values were aligned for direct comparison. Missing values in a specific channel were imputed as zero (indicating no detectable change). The differential regulation was calculated as the difference between the logFC in Channel H and Channel L. To determine the known biological significance of these sites, we annotated the phosphosites using the PhosphoSitePlus database.^42^

#### Motif enrichment and visualization analysis

Motif visualization analysis was performed using the pLogo generation tool,^48^ publicly available at http://plogo.uconn.edu/. As foreground, we used the sequence window of sites significantly up-regulated upon attack; as background, we used the whole identified phosphoproteome in the heavy channel (after the initial filtering steps). “No fixed position” was selected.

##### Kinase-substrate and pathway enrichment analysis

Kinase-substrate enrichment analysis was performed with RoKAi v2.3.0^51^ through the RoKAI App available at https://rokai.io/: fold Changes were set as “raw” and phosphatases were excluded from the analysis. The kinase table output was filtered to retain only kinases expressed on the proteome level, with adjusted p ≤ 0.05 and 2 or more substrates in at least one patient and channel.

Kinase-substrate enrichment analysis was also performed with PTM-Signature Enrichment Analysis (PTM-SEA),^47^ as implemented in PTMNavigator.^55^ To select kinases from the PTM-SEA output, only signature IDs containing the string “KINASE” were kept. Only kinases expressed on the proteome level and with adjusted p ≤ 0.05 in at least one patient and channel were used for the analysis.

Pathway enrichment analysis for phosphoproteomics data was performed with redundant single-sample Gene Set Enrichment Analysis (ssGSEA) 2.0, as implemented in PTMNavigator.^55^

For these analyses, we ranked sites using a signed -log10(*p*-value) metric derived from the Limma output. Specifically, for each phosphosite, the -log10 of the *p*-value was used if the corresponding log2 fold change was positive, while the log10 of the *p*-value (i.e., a negative value) was used if the fold change was negative. For the SILAC phosphoproteome data, phosphosites with the same residue and position but different multiplicity were collapsed to one value by keeping the maximum absolute value.

##### STRING network analysis

The functional protein network displayed in Figure 3C was generated in Cytoscape using STRING^87^ as implemented in the STRING app.^75^ The network was visualized using the Omics Visualizer app.^76^

##### CRISPR-KO screens

Data were downloaded from the original publications.^31^^,^^32^ In the Kearney et al. dataset, genes were considered enriched if *p* value was ≤ 0.01. In the Zhang et al. dataset, genes were considered enriched if fdr was ≤ 0.05.

##### Kaplan-Meier (KM) survival analysis

The KM curves were made with the Kaplan Meier plotter tool at https://kmplot.com/analysis/ using the immunotherapy tab. Tumor type was restricted to melanoma, while all the other settings were left unchanged.

##### Single-cell RNA sequencing (ssRNA-seq)

ssRNA-seq data^38^ were downloaded from the Single Cell Portal (https://singlecell.broadinstitute.org/single_cell/study/SCP109/melanoma-immunotherapy-resistance). Data was split in quartiles based on CRTAM gene expression. 0s were considered a separate group. Difference in the expression of selected genes between CRTAM quartiles was assessed by pairwise Wilcoxon Rank Sum. *p* values were adjusted with the Benjamini-Hochberg procedure.

##### TIDE (Tumor Immune Dysfunction and Exclusion) analysis

Genes of interest were queries on the “Query Gene” tab on the TIDE website (https://cide.ccr.cancer.gov/), and the data were downloaded from the “Expression” tab. Cancer types with less than 3 cohorts were excluded. Only overall survival (OS) data were used.

##### Data visualization

Plots were performed using the R package “ggplot2” version 3.5.1 or the GraphPad Prism software version 10. Euler diagrams were made through the R package “eulerr” version 7.0.2. Heatmaps were generated through the R package “pheatmap” version 1.0.12 or the R package “ggplot2” version 3.5.1. For all heatmaps, individual replicates were averaged after mean-centering or scaling, clustering, when performed, used Euclidean distance, unless specified otherwise.

## Supplemental information

Document S1. Figures S1–S7 and Table S2

Table S1. Significantly regulated phosphosites and pathways in SCC-25 cells stimulated with EGF and lysed immediately or after incubation on ice, related to Figure 1

Table S3. Significant proteomic alterations in melanoma cells and TILs upon 6-h T cell attack, related to Figure 3 and Figure 4

Table S4. Significant proteomic alterations in melanoma cells upon IFN-γ stimulation for 6 and 24 h, related to Figure 3

Table S5. Significant phosphoproteomic alterations in melanoma cells and TILs upon 2-h T cell attack, related to Figure 6

Table S6. Significantly regulated pathways and kinases in melanoma cells and TILs upon 2-h T cell attack, related to Figure 7

Table S7. HPLC, MS, and DIA-NN search parameters used in this paper, related to STAR Methods

Document S2. Article plus supplemental information
