# Supplementary material for: The proteomics and phosphoproteomics landscape of melanoma under T cell attack
Source: Cell Rep Med. 2026 May 21;7(6):102829. doi: 10.1016/j.xcrm.2026.102829 (PMC13293969; doi:10.1016/j.xcrm.2026.102829)
Supplement: Document S1. Figures S1–S7 and Table S2 [file mmc1.pdf]

Cell Reports Medicine, Volume *7*

Supplemental information

The proteomics and phosphoproteomics landscape of melanoma under T cell attack

Giulia Franciosa, Agnete W.P. Jensen, Ana Martinez-Val, Ilaria Piga, Marco Donia, and Jesper V. Olsen

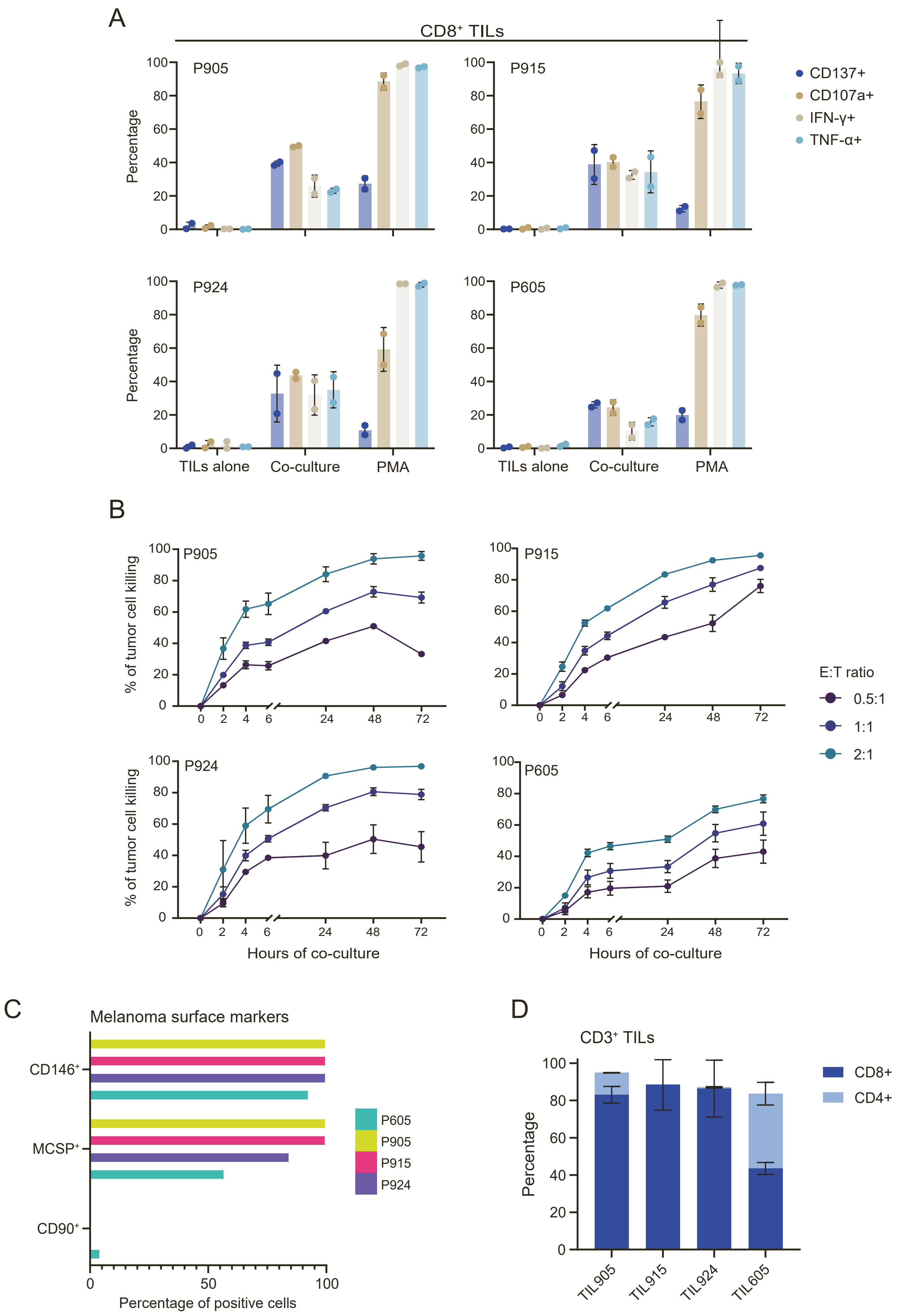

Figure S1. **Functional characterization of tumor-infiltrating lymphocytes (TILs) and melanoma patient-derived tumor cell lines (TCLs)**. Related to Figure 2. **A**. Flow cytometric analysis of tumor-specific CD8+ TIL reactivity based on upregulation of activation markers (CD137, CD107a) and intracellular cytokines (IFN-γ, TNF-α) after 8 hours of co-culture with autologous melanoma cells at an effector:target (E:T) ratio of 3:1. TILs alone and PMA/ionomycin stimulation served as negative and positive controls, respectively. Data are presented as mean ± SD (n=2 biological replicates). **B**. Autologous TIL-mediated killing of melanoma cells from four patients was assessed using xCELLigence real-time cell analysis at three different E:T ratios. The percentage of tumor cell killing was calculated as the Normalized Cell Index (NCI) of the tumor in co-culture divided by the NCI of tumor alone x 100. Data are shown as mean ± SD (n=3-4 technical replicates). **C**. Surface expression of two melanoma markers (CD146 and MCSP) and one fibroblast marker (CD90) in four different melanoma patient-derived TCLs. **D**. Surface expression of CD4 and CD8 by flow cytometry in CD3+ TILs. Data are presented as mean ± SD (n=2 biological replicates).

Figure S1

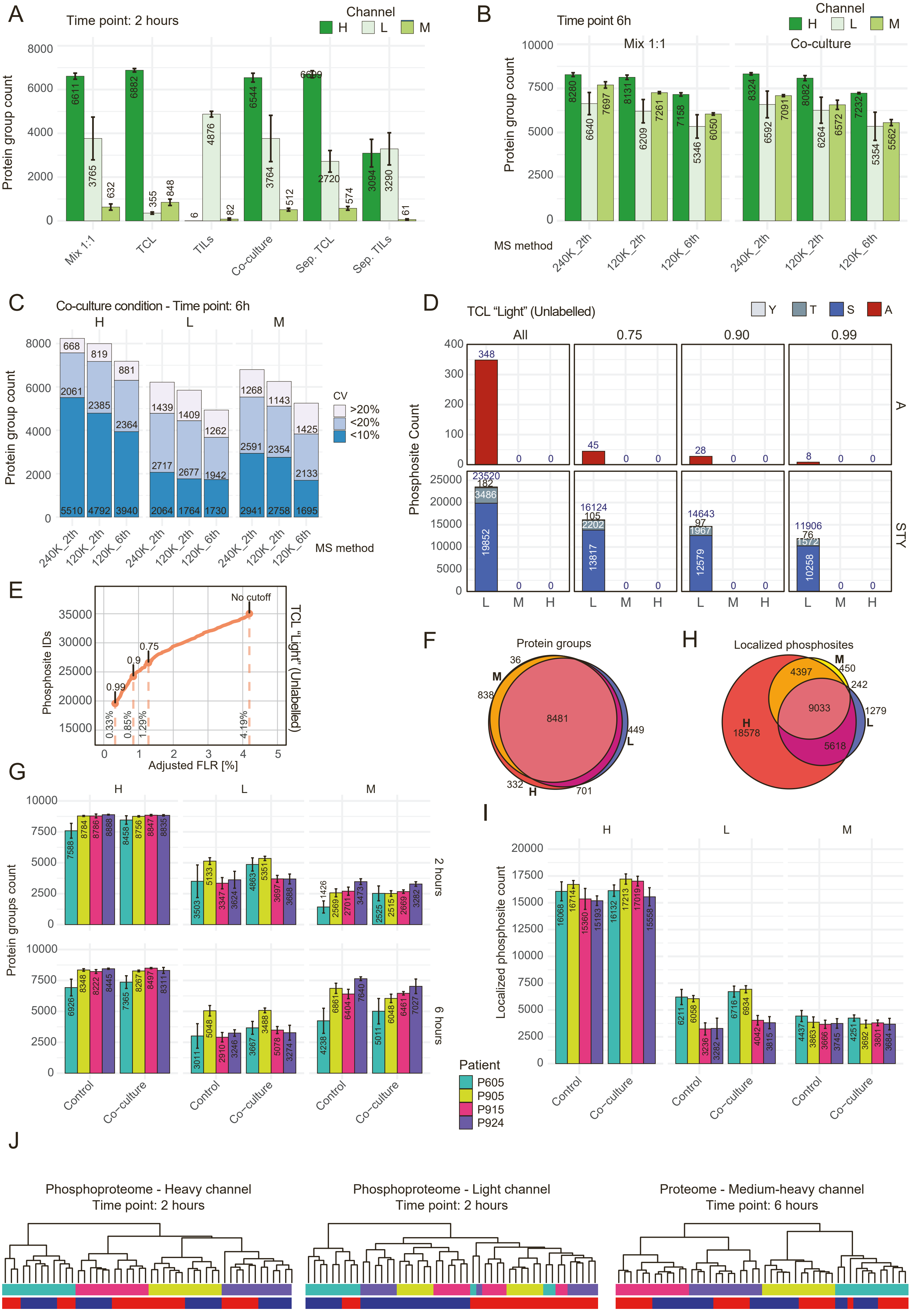

Figure S2

Figure S2. **SILAC-DIA proteome method optimization and coverage analysis**. Related to Figure 2. **A-C**. Pilot experiment results from patient 905. **A**. Protein group identifications per channel and condition after 2 hours of co-culture. Samples were analyzed on a 180 sample-per-day gradient. **B**. Protein group identifications per channel and MS method after 6 hours of co-culture, analyzed on a 36 sample-per-day gradient. Data in A and B are presented as mean ± SD (n = 4 biological replicates). **C**. Protein group coefficient of variation (CV) for the 6-hour co-culture condition (36 sample-per-day gradient), calculated for proteins with at least 3 out of 4 valid values. **D-E**. Phospho-Alanine Decoy Search to test reliability of localization algorithm. Analysis was performed on unlabeled ("light") phospho-enriched tumor samples (patient 924; n = 3). Alanine (A) serves as a negative control (decoy) to estimate the false localization rate (FLR). **D**. Stacked bar plots displaying phosphosite counts categorized by residue (S, T, Y, and A) across increasing localization probability thresholds (0, 0.75, 0.9 and 0.99). In the lower graph, numbers in blue represent the sum of S, T and Y. **E**. The number of localized target phosphosites is plotted against the adjusted False Localization Rate (FLR). FLR was calculated via a phospho-Alanine decoy entrapment search and adjusted for predicted residue frequencies. Dashed vertical lines denote specific localization probability cutoffs (0.75, 0.90, 0.99, and no cutoff), with the corresponding empirical FLR percentages labeled along the x-axis. **F-J**. Analysis of the four-patient cohort. **F**. Euler diagram displaying the overlap of protein group identifications across the three different channels. **G**. Protein group identifications stratified by channel, cell type, and patient at 2- and 6-hour time points. **H**. Euler diagram displaying the overlap of phosphosite identifications across the three channels. **I**. Phosphosite identifications stratified by channel, cell type and patient at 2- and 6-hour time points. Data in E and G are presented as mean ± SD (n = 6 biological replicates). **J**. Three separate dendrograms showing hierarchical clustering (Euclidean distance of Log2 MS intensities) for the phosphoproteome of the heavy channel, the phosphoproteome of the light channel, and the proteome of the medium-heavy channel at the 6-hour time point.

Figure S2

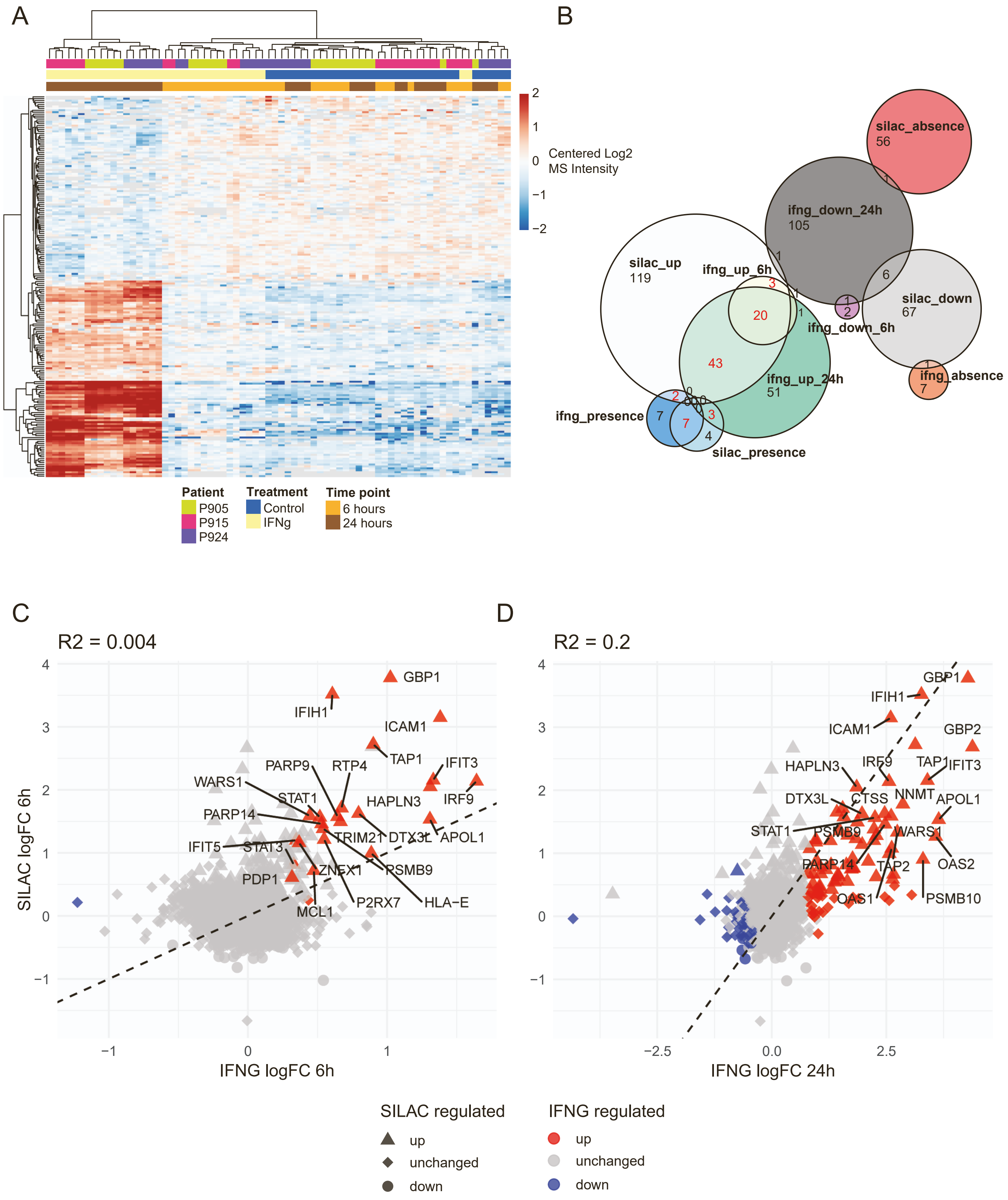

Figure S3. **Deconvolution of the IFN-γ-dependent proteome changes upon T cell attack**. Related to Figure 3. **A**. Heatmap of mean-centered log2 MS intensities for protein groups significantly regulated by IFN-γ in at least one time point and with more than 50% of valid values across the entire dataset (n=201 protein groups). Mean-centering was performed by patient. Both rows and columns were clustered with euclidean distance. **B**. Euler diagram showing the overlap between proteins regulated upon co-culture in the medium-heavy channel and by IFN-γ. Overlapping proteins coloured in red are represented in the STRING network displayed in Figure 3C. **C-D**. Scatter plots comparing protein fold-changes in the 6-hour co-culture (medium-heavy channel) versus IFN-γ treatment at 6 hours (C) and 24 hours (D). Labeled proteins are shared between the two datasets.

Figure S3

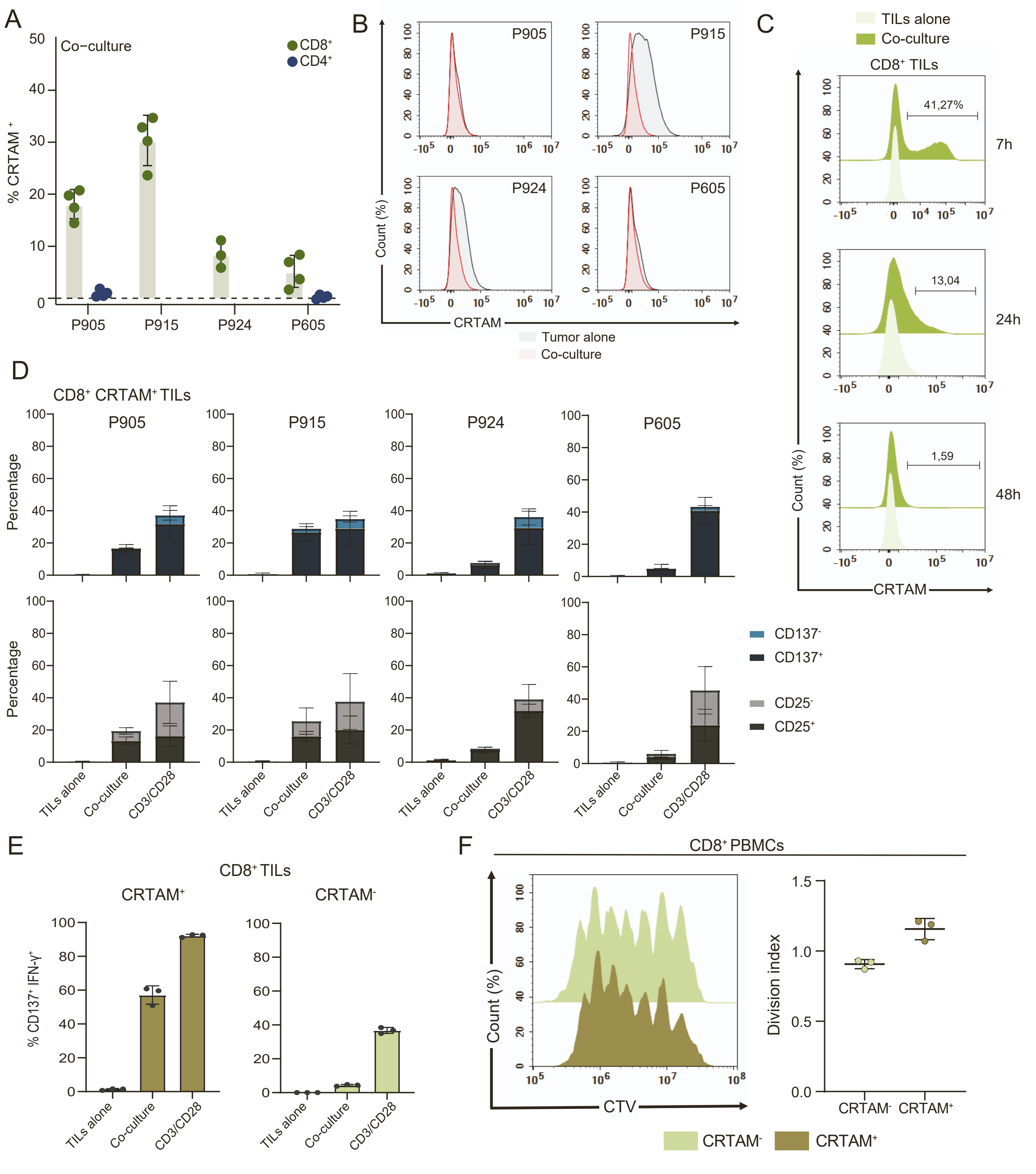

Figure S4. **CRTAM expression dynamics and association with T cell activation in melanoma co-cultures**. Related to Figure 5. **A**. Percentage of CRTAM+ cells within CD4+ and CD8+ TIL populations across all patients after 6 hours of co-culture with autologous melanoma cells. Note: CD4+ data are not shown for patients 915 and 924 due to negligible CD4+ counts in these REP pools. **B**. Histograms confirming the absence of CRTAM expression on melanoma cells, either cultured alone or after 6 hours of co-culture with autologous TILs. Data in A and B are presented as mean ± SD (n = 4 biological replicates). **C**. Time-course analysis of CRTAM expression (Patient 915) by flow cytometry analysis in patient CD8+ TILs after co-culture with autologous melanoma cells. **D**. Flow cytometric analysis of activation markers on CD8+ TILs. Plots show the proportion of CRTAM+ cells co-expressing CD137 (top) or CD25 (bottom) under three conditions: TILs alone, CD3/CD28 bead stimulation, and 6-hour autologous co-culture. Data are presented as mean ± SD (n = 2-4 biological replicates). **E**. Functional comparison of CRTAM+ versus CRTAM+ CD8+ TIL subsets (Patient 915). Bar graphs display the percentage of CD137+ FN-γ+ co-expression after 8 hours of stimulation with autologous tumor cells or CD3/CD28 beads. Data represent mean ± SD (n = 3 technical replicates). All co-culture experiments were performed at a 1:1 effector-to-target (E:T) ratio. **F**. PBMCs from healthy donors were labeled with CellTrace Violet (CTV) and stimulated with CD3/CD28 beads for 4 days, followed by flow cytometry analysis. Left panel: Representative CTV dilution histograms of stimulated CRTAM+ and CRTAM- CD8+ T cells. Right panel: Division index of stimulated CRTAM+ and CRTAM- CD8+ T cells. Data are presented as mean ± SD (n = 3 technical replicates). Dots represent individual replicates.

Figure S4

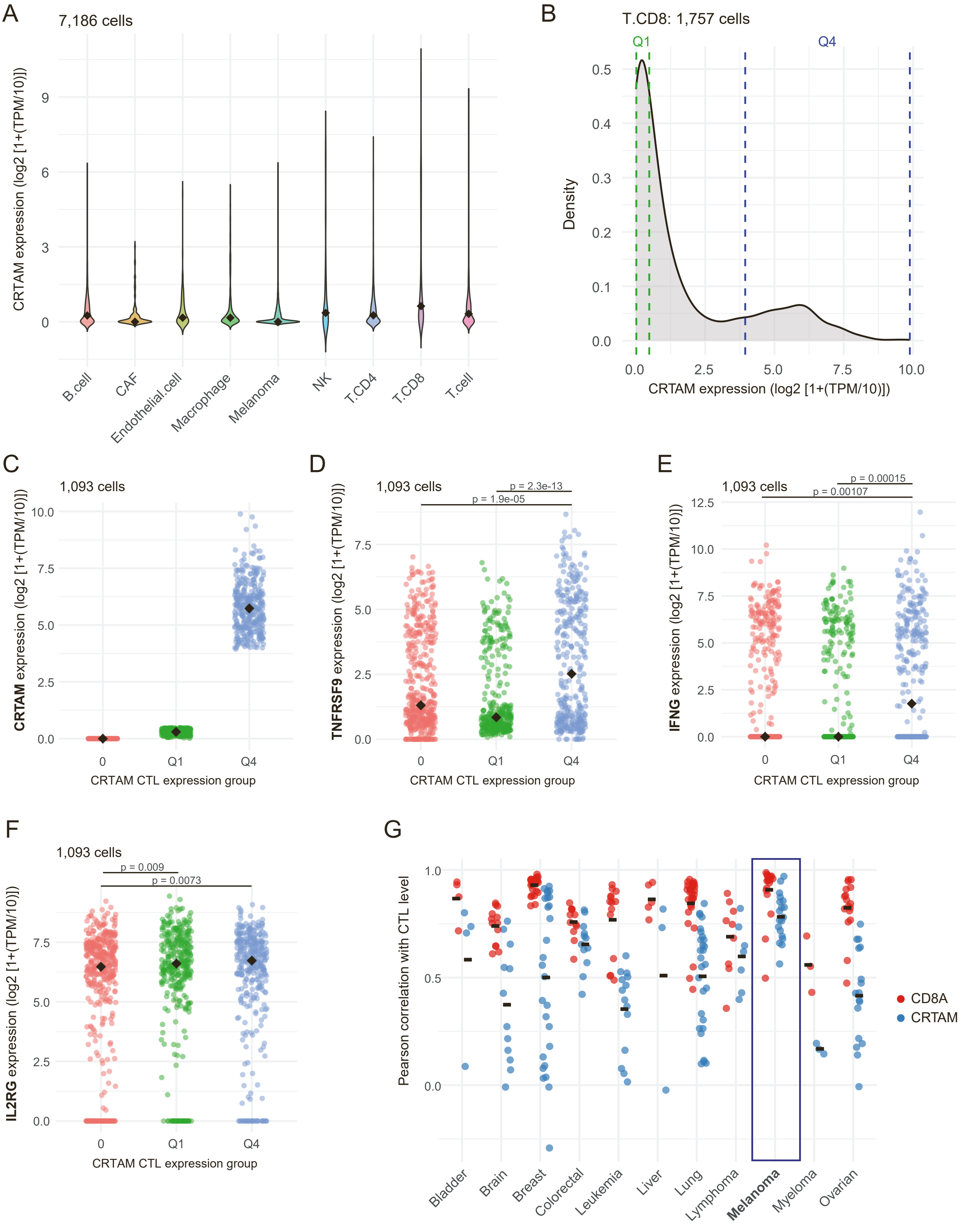

Figure S5. **Single-cell expression landscape of CRTAM and its association with cytotoxic T cell infiltration**. Related to Figure 5. **A**. Violin plot showing CRTAM gene expression at single-cell level across normal and malignant cell types in a melanoma scRNA-seq dataset. **B**. Histogram showing the CRTAM expression distribution at single cell level across CD8+ T cells. The first and fourth quartiles are coloured in green and blue, respectively. **C-F**. Expression levels of selected genes compared across CRTAM-negative (0), low-expressing (Q1), and high-expressing (Q4) CD8+ T cell subsets. p values were calculated by pairwise Wilcoxon Rank Sum Test and corrected with the Benjamini-Hochberg procedure. **G**. Analysis of the Tumor Immune Dysfunction and Exclusion (TIDE) database showing Pearson correlations between CRTAM or CD8A expression and cytotoxic T lymphocyte (CTL) infiltration levels across various cancer types.

Figure S5

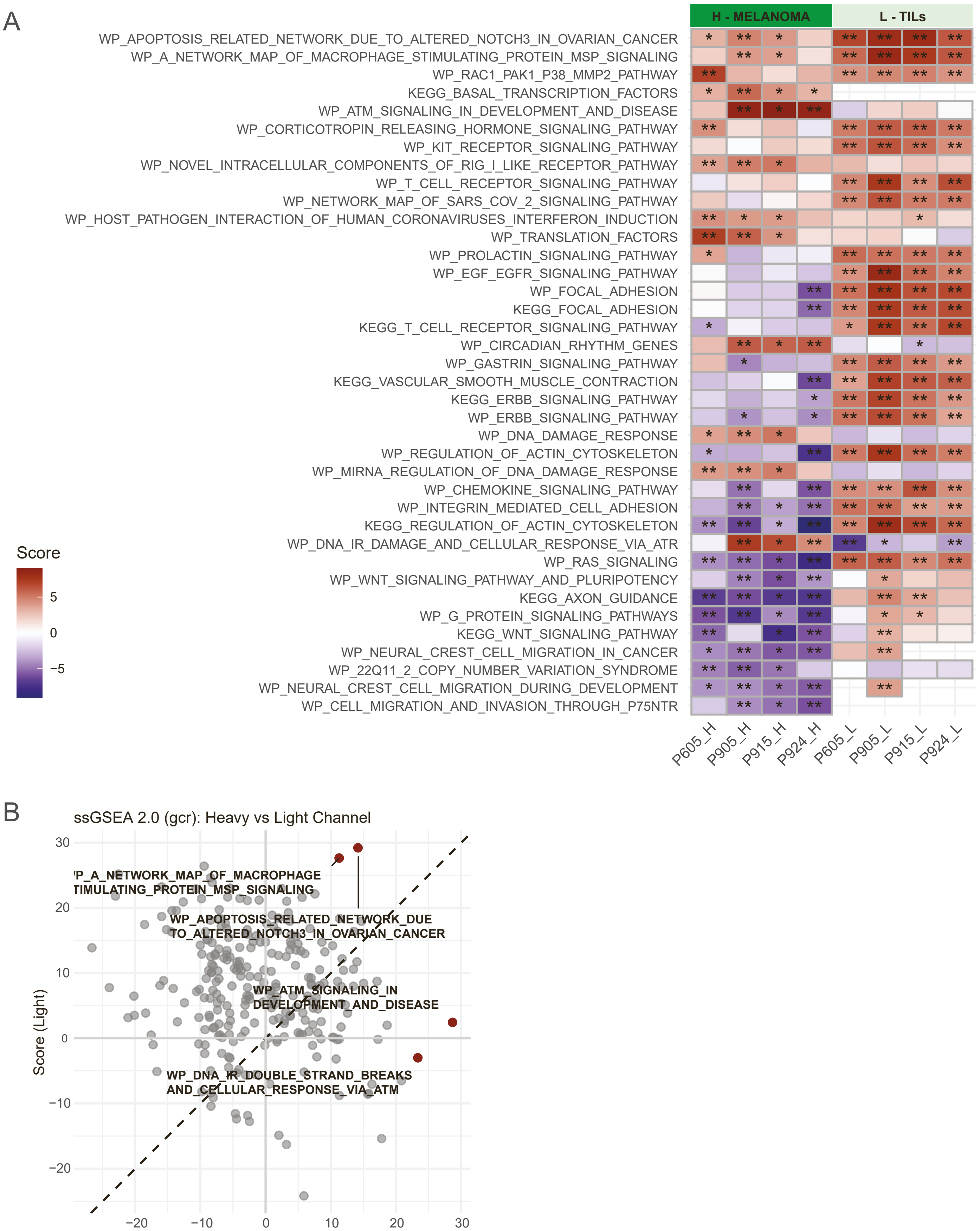

Figure S6. **Cell type-resolved analysis of phosphorylation driven-signaling pathways upon T cell attack**. Related to Figure 7. **A**. Heatmap of gene-centric-redundant ssGSEA scores. The 20 pathways with the highest sum of -log10(adjusted p) across the four patients per channel are shown, for a total of 38 unique pathways. Significance is represented by asterisks: * = adjusted p <= 0.05; ** = adjusted p <= 0.01; *** = adjusted p <= 0.001. **B**. Scatter plot of gene-centric-redundant ssGSEA scores in the heavy and light channels. The score is calculated by summing the individual patients' scores per channel. The two pathways with the highest score per channel are labeled. For both (A) and (B), only pathways with adjusted p <= 0.05 in at least one patient and channel are shown.

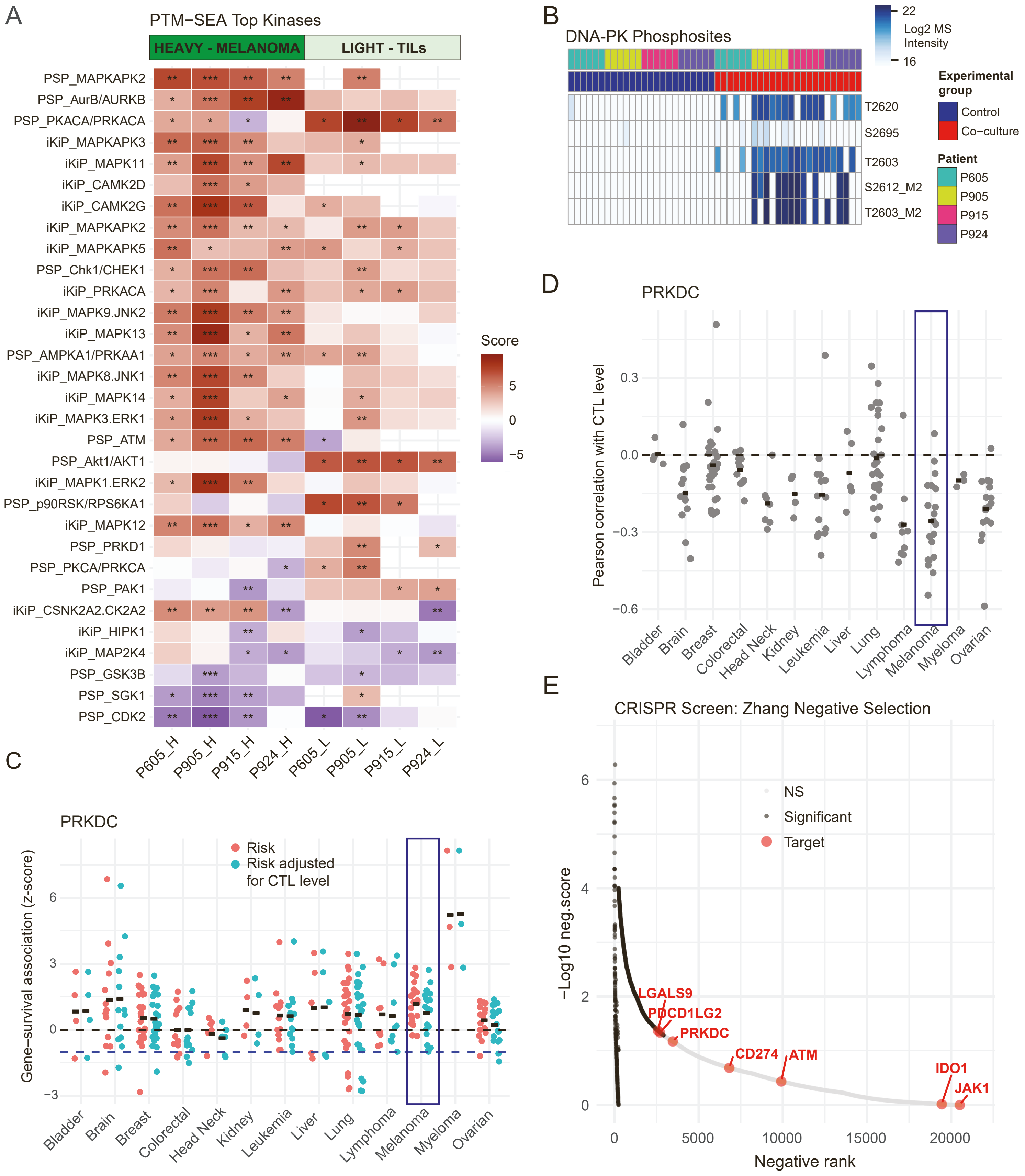

Figure S7. **Association of DNA-PK with melanoma immune evasion**. Related to Figure 7. **A**. Heatmaps of PTM-SEA kinase scores. Only kinases expressed on the proteome level and with adjusted p <= 0.05 in at least one patient and channel are shown. The 20 kinase signatures with the highest sum of -log10(adjusted p) across the four patients per channel were selected for this plot, for a total of 31 unique kinase signatures and 29 kinases. Significance is represented by asterisks: * = adjusted p <= 0.05; ** = adjusted p <= 0.01; *** = adjusted p <= 0.005. 7. **B**. Heatmap of log2 MS intensities of the 5 PRKDC phosphosites preferentially expressed upon co-culture, defined as having at most 2 out of 48 values in the control condition and at least 10 out of 48 in the co-culture condition. Missing values were imputed with a low value. **C**. Analysis of the Tumor Immune Dysfunction and Exclusion (TIDE) database showing Z-score of the effect of PRKDC gene expression on overall survival across multiple cancer types in a CoxPH model, before and after adjusting for CTL infiltration. A positive z-score means that higher PRKDC expression is associated with higher death risk. Melanoma cohorts are circled in blue. **D**. Analysis of the Tumor Immune Dysfunction and Exclusion (TIDE) database showing Pearson correlation between PRKDC gene expression and CTL infiltration across multiple cancer types. Melanoma cohorts are circled in blue. **E**. Negative score as a function of negative rank from the CRISPR-KO screen of Zhang et al. Genes with FDR <= 0.05 are colored in black. Some of the most well-known immune checkpoint molecules are labeled in red. JAK1 was also labeled as a reference, since it is supposed to display a phenotype opposite to the immune checkpoints.

	Cancer	Treatment before biopsis	Sex	Anti-PD1 (Naïve/Resistant)	Clinical response to clinical trial treatment
**P905**	Cutaneous metastatic melanoma	**1**. Nivolumab (anti-PD1), no response.	Male	R	Partial Response
**P915**	Cutaneous metastatic melanoma	**1**. Pembrolizumab (anti-PD-1), late response. Removed a remaining lesion.	Male	R	Not treated
**P924**	Cutaneous metastatic melanoma	**1**. IL-2, no response.<br>**2**. BRAFi, response and progression.	Male	N	Partial Response
**P605**	Cutaneous metastatic melanoma	No previous treatments	Male	N	Complete Response

Table S2. Clinical data from the 4 patients used in this study. Related to Figure 2.
